# Transient State Kinetics of *Plasmodium falciparum* Apicoplast DNA Polymerase Suggests the Involvement of Accessory Factors for Efficient and Accurate DNA Synthesis

**DOI:** 10.1021/acs.biochem.2c00446

**Published:** 2022-10-17

**Authors:** Anamika Kumari, Anjali Yadav, Indrajit Lahiri

**Affiliations:** † Department of Biological Sciences, Indian Institute of Science Education and Research Mohali, Punjab 140306, India; ‡ Molecular Microbiology, School of Biosciences, University of Sheffield, Sheffield S10 2TN, U.K.

## Abstract

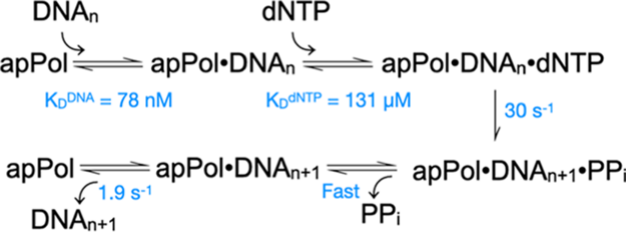

*Plasmodium*, the causative agent of malaria, belongs to the phylum Apicomplexa. Most apicomplexans, including *Plasmodium*, contain an essential nonphotosynthetic plastid called the apicoplast that harbors its own genome that is replicated by a dedicated organellar replisome. This replisome employs a single DNA polymerase (apPol), which is expected to perform both replicative and translesion synthesis. Unlike other replicative polymerases, no processivity factor for apPol has been identified. While preliminary structural and biochemical studies have provided an overall characterization of apPol, the kinetic mechanism of apPol’s activity remains unknown. We have used transient state methods to determine the kinetics of replicative and translesion synthesis by apPol and show that apPol has low processivity and efficiency while copying undamaged DNA. Moreover, while apPol can bypass oxidatively damaged lesions, the bypass is error-prone. Taken together, our results raise the following question—how does a polymerase with low processivity, efficiency, and fidelity (for translesion synthesis) faithfully replicate the apicoplast organellar DNA within the hostile environment of the human host? We hypothesize that interactions with putative components of the apicoplast replisome and/or an as-yet-undiscovered processivity factor transform apPol into an efficient and accurate enzyme.

## Introduction

*Plasmodium* is the causative agent of malaria, an infectious disease responsible for over 600,000 deaths per year.^1^ This pathogen belongs to the phylum Apicomplexa, and like most apicomplexans, *Plasmodium* contains a nonphotosynthetic plastid called the apicoplast, which is essential for the survival of the pathogen. In the blood stage of *Plasmodium*’s life cycle, this organelle acts as the site for isoprenoid precursor biosynthesis.^2^ Apicoplasts contain a small circular genome (apDNA) having repetitive A/T-rich sequence coding for apicoplast housekeeping genes.^3^ ApDNA replication initiates during the late trophozoite phase and continues into the schizogony phase of the parasite’s life cycle within the human red blood cells.^4^ This organellar genome is duplicated by a dedicated replisome with components that are divergent from other eukaryotic replisomes studied to date.^5^ At the heart of apicoplast replication is a polyprotein called PREX (plastidic DNA replication/repair enzyme complex). PREX is coded by the *Plasmodium* nuclear genome and contains primase, helicase, and DNA polymerase activities in a single polypeptide.^6^ After import into the apicoplast, PREX is proteolytically cleaved into the three known enzymatic components—the apicoplast primase, apicoplast helicase, and the apicoplast DNA polymerase (apPol).^7−9^

Both eukaryotic and prokaryotic cellular replisomes contain multiple DNA polymerases, each with a specialized function. For instance, in the bacterial replisome, the α-subunit of the DNA polymerase III holoenzyme copies the majority of the genome (replicative synthesis) with high speed and accuracy, while DNA polymerases II, IV, and V are involved in duplicating the damaged portions of the bacterial DNA (translesion synthesis, TLS).^10^ In contrast, apPol is the only DNA polymerase identified in the apicoplast to date and forms the core enzymatic component of its replisome.^5^ One possible scenario is that apPol performs the functions of multiple specialized DNA polymerases including replicative and translesion synthesis. However, both the kinetic and structural mechanisms used by the apicoplast replisome to accurately duplicate apDNA with help from a single DNA polymerase remain unresolved.

**Figure 1:**
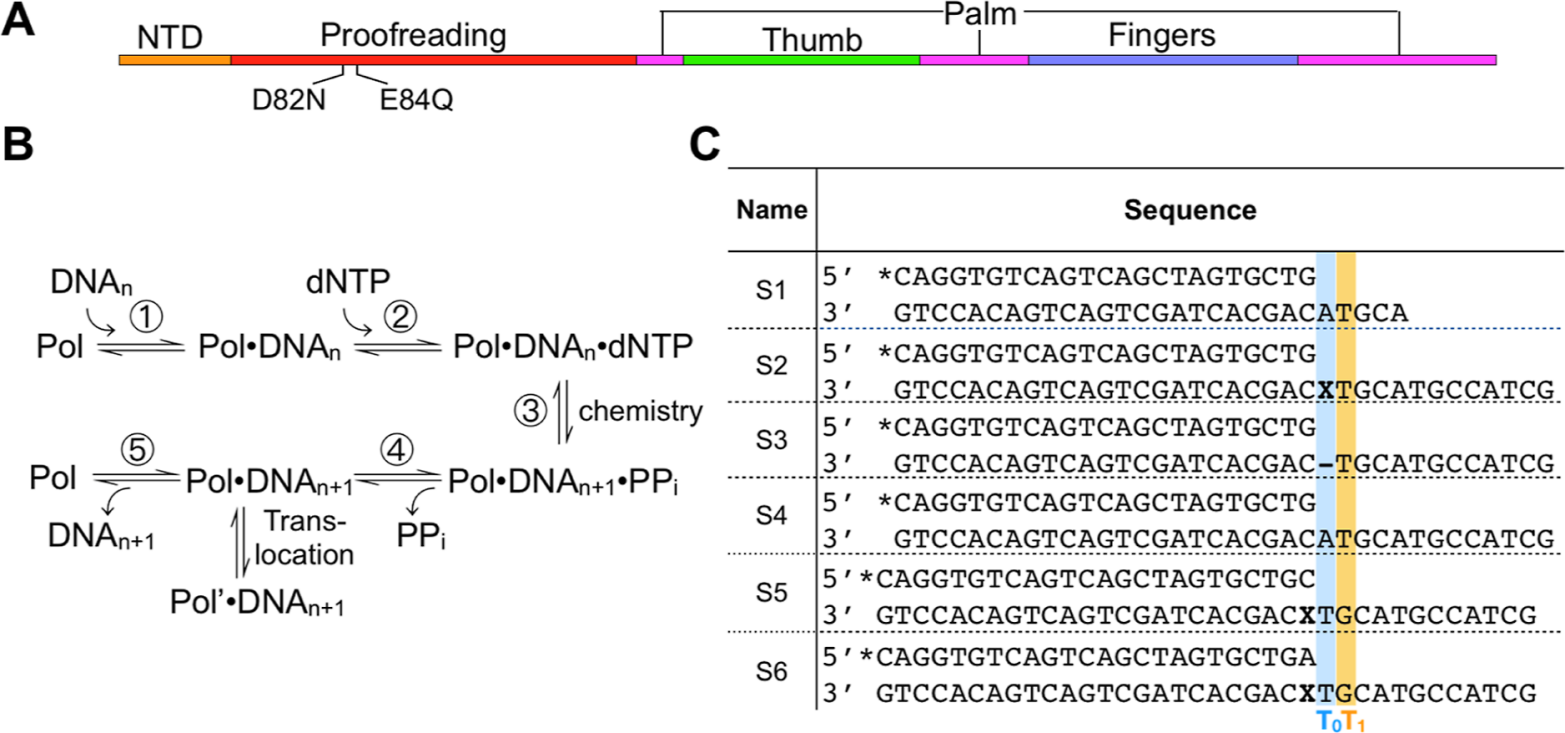
apPol domain organization, the kinetic pathway of nucleotide incorporation by DNA polymerases and DNA substrates used in primer extension assays. (A) Domain organization of *P. falciparum* apPol. Domain boundaries are based on the crystal structure of apPol, and the two residues (D82 and E84) of the proofreading exonuclease domain that were mutated (to N and Q, respectively) to eliminate the exonuclease activity are highlighted. NTD: N-terminal domain (orange), proofreading: 3′ to 5′ proofreading exonuclease domain (red). The palm, thumb, and fingers domains are colored in magenta, green, and blue, respectively. (B) Minimal kinetic pathway for nucleotide incorporation by a DNA polymerase (Pol), where DNA_*n*_: DNA substrate with a primer strand n bases long, DNA_n+1_: DNA substrate with a primer strand n+1 bases long, PP_i_: inorganic pyrophosphate, and Pol’: DNA polymerase translocated by one base. (C) DNA substrates used to define the polymerization pathway of apPol. The templating base positions (T_0_) and the one immediately 5′ to it (T_1_) are shaded blue and orange, respectively. The oxidatively damaged nucleotides (ODNs) are highlighted in bold and represented as **X**: 8-oxo-7,8-dihydroguanosine monophosphate (8-oxo-dGMP) and—: tetrahydrofuran apurinic/apyrimidinic site (abasic site) analogue. “*” represents the 5′FAM label.

apPol belongs to the A-family of DNA polymerases, and within the A-family apPol is placed in a poorly studied clade of viral DNA polymerases.^11^ Apoenzyme structures of apPol provide an overview of this enzyme’s architecture.^12,13^ The overall structure of apPol resembles that of a typical DNA polymerase with two active sites, one for the polymerization activity and the other for 3′ to 5′ exonuclease proofreading activity. apPol does not have a 5′ to 3′ exonuclease domain. Instead, it has been postulated that a separate protein containing the 5′ to 3′ exonuclease activity acts *in trans* with apPol.^5^ apPol has an additional N-terminal domain (NTD) of unknown functions (Figure 1A). The structural information has been complemented with preliminary functional characterizations of apPol using steady-state multiple-turnover kinetics.^8,14,15^

All DNA polymerases, with a few exceptions,^16,17^ follow the same sequence of events when incorporating a deoxynucleoside triphosphate (dNTP) into a growing DNA strand (Figure 1B). Different polymerases achieve their unique catalytic signatures, required for their specialized functions, by virtue of altered rate constants governing the individual steps of the enzymatic cycle. In the first step, the DNA polymerase and DNA substrate form a prechemistry binary complex (Pol·DNA_*n*_). The incoming dNTP then binds to the binary complex to form the prechemistry ternary complex (Pol·DNA_*n*_·dNTP) followed by polymerase-mediated catalysis of the nucleotidyl transfer reaction resulting in the incorporation of the incoming dNTP into the primer strand, thus elongating the DNA primer by a single nucleotide (DNA_*n*+1_). This chemistry step is often preceded by one or more steps through which the ground-state prechemistry ternary complex converts to the active state, poised for catalysis. Following primer extension, the pyrophosphate(PP_i_) byproduct dissociates from the postchemistry ternary complex (Pol·DNA_*n*+1_·PP_i_) leading to a postchemistry binary complex (Pol·DNA_*n*+1_). In the case of single nucleotide addition, the polymerase will dissociate from the extended DNA, and the cycle will be repeated. For processive synthesis, instead of dissociating, the polymerase translocates along the DNA (Pol′·DNA_*n*+1_) to the next templating position, and the cycle is repeated.

While some preliminary kinetic characterization of apPol performed under multiple turnover conditions has been reported, these assays do not provide an accurate picture of the catalytic cycle of a DNA polymerase,^18^ and to date, the kinetic mechanism of replication by apPol remains unknown. To address this gap, we have used transient state kinetics to build a comprehensive picture of the enzymatic mechanism of apPol. We find that apPol catalyzes DNA extension with low efficiency and processivity, properties that would prevent this polymerase from performing replicative synthesis. Notably, we show that while apPol can perform robust TLS against oxidatively damaged nucleotides (ODNs), this bypass is error-prone and apPol’s intrinsic proofreading activity does not preferentially excise out the misincorporated nucleotide. Our work lays down a foundation for understanding the mechanism of organellar genome duplication in *Plasmodium* apicoplast, a fundamental process for pathogen survival.

## Experimental Procedures

### DNA Substrates

All undamaged DNA oligonucleotides were purchased from Integrated DNA Technology (USA). Oxidative damage-containing DNA oligonucleotides were purchased from TriLink Biotechnologies (USA). DNA substrates were generated by annealing the primer and template DNA strands as shown in Figure 1C. All annealing reactions were performed using a 1:1.1 ratio of primer and template DNA in an annealing buffer (10 mM Tris-Cl (pH 7.5) and 50 mM NaCl). The sample was heated to 95 °C for 2 min, followed by gradual cooling to 25 °C.

### Software

All gels were analyzed using ImageQuant TL version 10.1 (Cytiva, USA). Graph plotting and nonlinear regressions were performed using Prism version 9.3.1 (Graphpad Software, USA). Global fitting of the primer extension data using numerical integration was performed using KinTek Explorer version 10.2.3 (KinTek Corp., USA).

### apPol Constructs

For all the experiments except the exonuclease assays, we have used a construct of apPol harboring two point mutations (D82N and E84Q) at the active site of the proofreading exonuclease domain (Figure 1A). The mutant construct (referred to as apPol) showed no detectable DNA degradation (data not shown) and allowed us to focus on the polymerization activity. For the exonuclease assays, the wild-type apPol (apPol^WT^) lacking the two point mutations mentioned above has been used.

### Cloning, Overexpression, and Purification of apPol

Both apPol and apPol^WT^ were cloned, overexpressed, and purified following similar strategies. A gene construct of *Plasmodium falciparum* apicoplast DNA polymerase containing an N-terminal hexa-histidine tag followed by a tobacco etch virus (TEV) protease cleavage site, codon optimized for expression in *Escherichia coli,* was synthesized and cloned into the pETDuet1 vector by GenScript Corp. (USA).

The plasmid was transformed into Rosetta 2(DE3) *E. coli* cells (Merck, USA). *E. coli* cells were grown in autoinduction terrific broth at 37 °C for ∼5 h, and then, growth was maintained at 20 °C for ∼20 h before harvesting the cells by centrifugation. All subsequent steps were carried out at 4 °C. Cell pellets were resuspended in lysis buffer containing 50 mM Tris–HCl (pH 7.5), 800 mM NaCl, 25 mM imidazole, and 10% glycerol. To prevent proteolytic degradation of apPol and improve lysis efficiency, ethylenediaminetetraacetic acid (EDTA)-free protease inhibitor tablet (Roche, USA) and lysozyme were added to the resuspended cells. Cells were lysed by sonication and clarified by centrifugation. The clarified lysate was loaded onto a 5 ml HiTrap Chelating HP column (Cytiva, USA) charged with Ni^2+^ and pre-equilibrated with lysis buffer. The unbound protein was washed with 10 column volumes (CVs) of lysis buffer, followed by an additional 10 CV wash with low-salt wash buffer [50 mM Tris–HCl (pH 7.5), 200 mM NaCl, 25 mM imidazole, and 10% glycerol]. apPol was eluted over a 10 CV linear gradient of imidazole from 25 mM to 1 M. The elution factions were analyzed using SDS-PAGE, and fractions containing apPol were pooled and diluted with no-salt buffer [50 mM Tris–HCl (pH 7.5), 25 mM imidazole, and 10% glycerol] to decrease the NaCl concentration to 100 mM. The diluted sample was loaded on a 5 ml HiTrap SP column (Cytiva, USA) pre-equilibrated with Buffer A [50 mM Tris–HCl (pH 7.5), 100 mM NaCl, 5 mM EDTA, 5 mM β-mercaptoethanol, and 10% glycerol]. Unbound protein was washed with 10 CVs of Buffer A, and apPol was eluted with a linear gradient of 0.1 to 1 M NaCl (10 CVs). For further purification, the eluent of the S-column was concentrated and loaded on a Superdex 200 Increase 10/300 size-exclusion chromatography column (Cytiva, USA) pre-equilibrated with storage buffer [50 mM Tris-Cl (pH 7.5), 200 mM NaCl, 2 mM DTT, and 20% glycerol]. Fractions containing pure apPol were pooled, concentrated, flash-frozen in liquid nitrogen, and stored at −80 °C. Protein concentration was determined based on the theoretical extinction coefficient of 64180 M^–1^ cm^–1^. The hexa-histidine tag was not removed prior to using the protein.

### Single Nucleotide Primer Extension Assays

Primer extension assays were performed at 37 °C in reaction buffer containing 20 mM Tris-Cl (pH 7.5), 10 mM MgCl_2_, 30 mM NaCl, 1 mM DTT, 0.1 mg/mL BSA, and 5% glycerol. The assays were set up under burst conditions such that there was a slight excess of the DNA substrate after preforming the prechemistry binary complex. A final concentration of 480 nM (104 nM was active) apPol was incubated with various DNA and nucleotide substrates as mentioned in the corresponding figure legends. The active fraction of apPol was estimated as described in the “Results” section (Figure S1). The reactions were quenched using 250 mM EDTA and analyzed on 15% polyacrylamide (19:1) urea (7.5 M) gels run at 45–50 °C. The gels were imaged on a Typhoon FLA7000 LASER-based scanner (Cytiva, USA) using an excitation wavelength of 488 nm (blue LASER) and an emission cutoff at 525 nm. This allowed the detection of the fluorescence signal from the FAM-labeled primer DNA (Figure 1C, top strands). The gel bands were quantitated using Image Quant software (Cytiva, USA), and the concentration of extended DNA was calculated using the following formula.
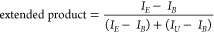
1where *I*_E_ is the intensity of the extended primer band, *I*_B_ is the intensity of the gel background, and *I*_U_ is the intensity of the unextended primer band.

All primer extension assays were performed using an RQF-3 rapid quench instrument (KinTek Corp., USA) except those with DNA substrate S3, which were performed by manual mixing of the reagents. The biphasic time courses were fit to the full burst equation
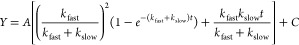
2where *Y* is the product concentration, *A* is the amplitude of the fast phase, *k*_fast_ and *k*_slow_ are the rates for the fast and slow phases, respectively, *t* is the time interval, and *C* is a constant.

Time courses that did not show a biphasic behavior were fit to the single exponential equation.
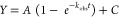
3where *Y* is the product concentration, *A* is the amplitude, *k*_obs_ is the rate of product formation, *t* is the time interval, and *C* is a constant.

To determine the binding affinity of apPol for its DNA substrate, primer extension assays were performed with a final concentration of 480 nM apPol, 500 μM dTTP, and varying concentrations of DNA substrate S1 (50, 100, 200, 400, and 800 nM). The reactions were incubated for different time intervals (0 to 0.8 s) and quenched with EDTA. Product concentrations were plotted as a function of time, and data were fit to a full burst equation. The amplitudes calculated from the time courses were plotted as a function of DNA concentration, and the data were fit to the quadratic equation
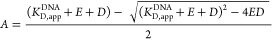
4where A is the amplitude, *K*_D,app_^DNA^ is the apparent equilibrium dissociation constant of the apPol·DNA binary complex, *E* is the active apPol concentration, and *D* is the DNA concentration.

To assess the rate of nucleotide incorporation (*k*_pol_) and the apparent equilibrium dissociation constant (*K*_D,app_^dNTP^) of the nucleotide for the prechemistry binary complex, primer extension assays were performed under the burst condition by incubating final concentrations of the 104 nM active apPol and 100 nM DNA (the identity of the DNA substrate varied between S1, S2, and S3 depending on the experiment) and varying concentrations of the incoming dNTP. The reactions were incubated at 37 °C at varying time intervals and then quenched with EDTA. The time intervals were varied depending on the identity of the DNA and are mentioned in the respective figure legends. Extended products were plotted as a function of time, and data were fitted to either the burst equation (eq 2) or the single exponential equation (eq 3). k_fast_ (for biphasic time courses) or k_obs_ (for single exponential time courses) was plotted as a function of dNTP concentration, and the data were fit to the hyperbolic equation
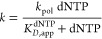
5where k is either *k*_fast_ or *k*_obs_ (depending on the nature of the time course), *k*_pol_ is the maximal rate of polymerization, *K*_D,app_^dNTP^ is the apparent dissociation constant of nucleotide binding, and dNTP is the nucleotide concentration.

### Double Mixing Experiment to Determine the Binary Complex Dissociation Rate

To measure the rate of DNA dissociation from the binary complex, a preincubated mixture of 208 nM active apPol and 400 nM DNA substrate S1 was mixed with an equal volume of 100 μM trap DNA (S1 without the FAM label) such that the final concentrations of active apPol, S1, and trap were 104 nM, 200 nM, and 50 μM, respectively. The mixture was incubated for various time intervals ranging from 0 to 6 s. This was followed by the addition of 500 μM dTTP and a second incubation for 170 ms before being quenched with excess EDTA. The product concentration was plotted as a function of the first incubation time, and data was fit by global fitting.

### Multiple Nucleotide Primer Extension Assay

Multiple nucleotide primer extension assays were performed and analyzed using a protocol similar to the one for single nucleotide primer extension assays with a few differences as mentioned below.

To calculate the average rate of processive synthesis, primer extension assays were performed with a final concentration of 104 nM active apPol; 200 nM DNA substrate S1; 250 μM each of dTTP, dATP, and dCTP; and 50 μM trap DNA (unlabeled S1). Reactions were incubated at 37°C for different time intervals of 0 to 4 s before quenching with excess EDTA. Products of three consecutive nucleotide additions were plotted as a function of time, and data were analyzed by global fitting using the KinTek Explorer.

To assess the lesion bypass capability of apPol against oxidative damages, multiple nucleotide extension assays were performed using a final concentration of 104 nM active apPol, 200 nM DNA substrate (S2, S3, or S4), and 250 μM of each of the four dNTPs. Reactions were incubated at 37°C for 0 to 20 min and then quenched with excess EDTA. Extended products were analyzed on 15% acrylamide-urea gel.

### Primer Degradation Assay

Primer degradation assays were performed to investigate apPol’s pyrophosphorolysis and 3′ to 5′ proofreading exonuclease activities. For assessing pyrophosphorolysis, a final concentration of 104 nM active apPol and 100 nM DNA substrate S1 was incubated with varying concentrations of inorganic pyrophosphate (2000, 1000, 500, 250, and 125 μM) in apPol reaction buffer for 0 to 2 min followed by quenching with EDTA, and for assessing the proofreading activity, final concentrations of 104 nM wild-type apPol (apPol^WT^) and 200 nM DNA substrate (S7, S8, S9, or S10) were incubated with a final concentration of 10 mM MgCl_2_ for 0 to 30 s before quenching with EDTA. Degraded products were analyzed on a 15% acrylamide-urea gel, and the concentration of the shortened primer (by a single nucleotide) was plotted as a function of time. The time course was fit to the burst equation, and the rate of the fast exponential phase (*k*_fast_) was approximated as the exonuclease rate (*k*_exo_).
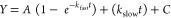
6where *Y* is the product concentration, *A* is the amplitude of the fast phase, *k*_fast_ is the rate of product formation for the fast phase, *k*_slow_ is the rate of product formation for the slow phase, *t* is the time interval, and *C* is a constant.

### Phosphate Release Assay

M13mp18 single-stranded DNA was annealed to a 75-nucleotide-long primer. A final concentration of 10 nM annealed M13mp18 DNA was incubated with 104 nM of active apPol and 250 μM of each of the four dNTPs. The reaction was incubated at 37°C for 30 min and then quenched with 125 mM EDTA (final concentration). 50 μL of the quenched assay was added to 100 μL of a proprietary malachite green solution (MilliporeSigma, USA). The sample was then transferred to a clear 96-well plate, and the absorbance was measured at 620 nm (A_620_). The absorbance was converted to the amount of phosphate released by comparing the A_620_ value to a standard curve prepared using the inorganic phosphate (P_i_) standard solution supplied with the malachite green following the manufacturer’s protocol. A control reaction to determine the amount of phosphate present in the buffer was performed using a similar protocol with the following exception. In this case, EDTA was added to the reaction mix prior to adding the nucleotides. Since EDTA was added before the dNTPs, no polymerization reaction could take place.

### Determination of Kinetic Constants from Data Fitting Using Numerical Integration

Global fitting of the kinetic data using numerical integration was performed using the KinTek Explorer.^19^ The kinetic pathways used for data fitting are described in the “Results” section. In all the cases, the association rate constant governing the binding of the incoming dNTP to the prechemistry binary complex was locked at a diffusion-limited macromolecular association rate constant of 10 μM^–1^s^–1^.^20^ We performed one-dimensional and two-dimensional confidence contour analyses using the FitSpace function in KinTek Explorer.^21^ The chi square threshold suggested by the software was used to calculate the 95% confidence interval.

While fitting the primer extension data for the abasic site bypass, we noticed that for the second dATP addition, the rate constant governing the second chemistry step and the K_D_^dATP^_2_^nd^_addn._ could not be independently determined with high precision. However, the efficiency of the second addition given by the ratio of these two kinetic constants (rate constant governing second chemistry/*K*_D_^dATP^_2_^nd^_addn._) was well constrained by our data. Accordingly, these two parameters were linked at their best fit values during global fitting and one-dimensional (1D) and two-dimensional (2D) confidence contours.

## Results

### Kinetic Mechanism of Correct Nucleotide Incorporation by apPol

We have determined the kinetic mechanism used by apPol for cognate nucleotide incorporation opposite an undamaged base. We performed transient state kinetic assays with comparable apPol and DNA concentrations which allowed us to define the individual steps of apPol’s catalytic cycle. The time courses of product formation were analyzed by global fitting through numerical integration. Traditional data fitting approaches using nonlinear regression provide a wealth of information about the individual steps of the catalytic cycle; however, this type of analysis requires several simplifying assumptions in order to find general solutions to the rate equations and the intrinsic relations between the observables from separate experiments might often be ignored. To overcome these limitations, we analyzed our primer extension data using KinTek Explorer software, which allowed us to fit all the experimental results by numerical integration to a global kinetic model. Based on the global fit, we have defined the rate constants governing the minimal catalytic cycle for correct nucleotide incorporation by apPol.

### Phosphodiester Bond Formation Is Followed by Slow Dissociation of the Product DNA

**Figure 2:**
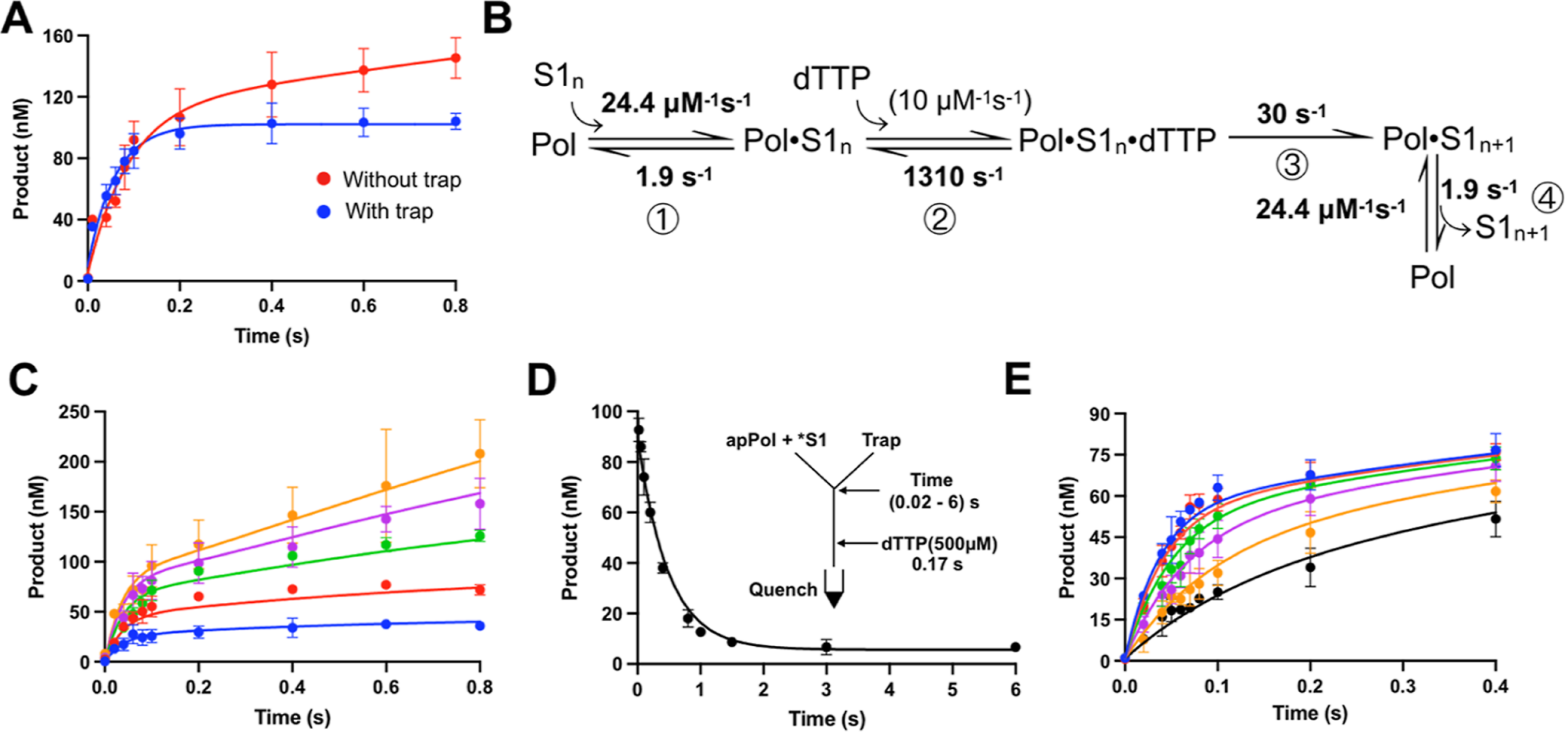
Correct nucleotide incorporation by apPol opposite to an undamaged template. (A) Time courses of product formation from single nucleotide primer extension assays with DNA substrate S1 and dTTP as the incoming nucleotide in the presence (blue) and absence (red) of trap DNA (S1 without the 5′ FAM label). The reaction was set at 37 °C under a presteady-state burst condition with final concentrations of 200 nM DNA, 480 nM apPol (104 nM is active; see figure legend for (C)), and 500 μM dTTP. The reactions were quenched at various time points ranging from 0 to 0.8 s using 250 mM EDTA. The product formed was plotted as a function of time and fitted to the full burst equation (without trap) or the single exponential equation (with trap). (B) Kinetic model used for global fitting. The best fit values of the rate constants governing the different steps are highlighted in bold. The rate constants in parenthesis were not allowed to vary during global fitting. S1_*n*_: DNA substrate S1 with unextended 23 nucleotides long primer strand. S1_n+1_: S1 with a primer strand extended by a single nucleotide. (C) Active site titration of apPol. Single nucleotide primer extension assays were set up as described for (A) with the following changes. No trap DNA was added to the assay, and the final concentration of the DNA substrate S1 was varied from 50 to 800 nM (50 nM: blue, 100 nM: red, 200 nM: green, 400 nM: magenta, and 800 nM: orange). The time courses of product formation were fit by numerical integration. K_D_^DNA^ was determined to be 77.9 nM. (D) Determination of the dissociation rate of the prechemistry binary complex. 400 nM DNA substrate S1 was preincubated with 208 nM active apPol. This sample was incubated with an equal volume of 100 μM trap DNA (S1 without the FAM label) for various time intervals ranging from 0.02 to 6 s. Therefore, the final concentrations of active apPol, S1, and trap DNA were 104 nM, 200 nM, and 50 μM, respectively. After incubation, an equal volume of 1 mM dTTP was added to the reaction and incubated for another 0.17 s. The reaction was quenched with 250 mM EDTA, and the sample was analyzed on a denature acrylamide-urea gel. The product formed (substrate S1 extended by one nucleotide) was plotted as a function of the first incubation time, and the data were fit by numerical integration. Based on the fit, the rate constant governing dissociation of the prechemistry binary complex (k_–1_) was calculated to be 1.9 s^–1^. **Inset**: Schematic of the experimental setup. *: FAM label. (E) Primer extension assays for determination of the rate of polymerization and the affinity of dTTP for the prechemistry binary complex (*K*_D_^dNTP^). Single nucleotide primer extension assays we set up as described for (A) with the following changes. No trap DNA was added to the assay, and the final concentration of dTTP was varied from 15.6 to 500 μM (15.6 μM: black, 31.25 μM: orange, 62.5 μM: magenta, 125 μM: green, 250 μM: red, and 500 μM: blue), and the incubation time ranged from 0 to 0.4 s. The time courses of product formation were fit by numerical integration, and *K*_D_^dNTP^ was 131 μM, while k_3_, the rate constant governing the chemistry step, was 30 s^–1^. All the experiments were performed in triplicate, and the average of the three independent data sets is plotted in the graphs shown in (C–E), while the error bars represent the standard deviation (SD) of data sets. The smooth lines overlaying the data represent the best fit based on global fitting.

We performed a single nucleotide primer extension assay using DNA substrate S1 (Figure 1C) under the presteady-state burst condition. Such an assay allowed us to monitor the kinetics of the first as well as subsequent rounds of primer extension. We observed a biphasic time course of nucleotide incorporation and fit the data to the full burst equation (eq 2) with a fast exponential phase (*k*_fast_ = 11.2 ± 2.9 s^–1^), followed by a slower linear phase (*k*_slow_ = 0.37 ± 0.3 s^–1^) (Figure 2A, red curve). Our observation can be explained by a preformed apPol·DNA binary complex rapidly forming the elongated product (Figure 1B, DNA_n+1_) in the first round of catalysis, followed by a slow postchemistry step that would limit the rate of subsequent turnover of apPol and result in the slow linear phase. Such biphasic time courses have been observed for nearly all DNA polymerases owing to the general paradigm that the chemical step of bond formation is followed by a slower step.

While the biphasic nature of product formation indicates the presence of a slow postchemistry step, it does not identify the rate-limiting step. For most DNA polymerases, dissociation of the postchemistry binary complex (Figure 1B, step 5) dominates the slow linear phase. To determine whether product DNA release was the slow step in apPol’s catalytic cycle, we performed primer extension under burst conditions but added an excess of unlabeled trap DNA (substrate S1 without the FAM label) along with the incoming dTTP. As before, the prebound labeled DNA would rapidly convert to the product, but upon dissociation from the postchemistry binary complex (apPol·DNA_*n*+1_), apPol would be “trapped” by the excess unlabeled DNA preventing any subsequent rounds of catalysis from being detected. We found that in the presence of a trap, the slow phase is absent (Figure 2A, blue curve), indicating that dissociation of the postchemistry binary complex is indeed the rate-limiting step in apPol’s catalytic cycle. The rate of product formation was 16.9 ± 1.9 s^–1^, in good agreement with *k*_fast_ in the absence of a trap.

### apPol Binds Weakly to DNA Due to Rapid Dissociation of the Binary Complex

Replicative DNA polymerases that copy the bulk of a genome need to remain bound to the substrate DNA through multiple cycles of polymerization which translates to a high affinity for the DNA. A-family-replicative polymerases such as the bacteriophage T7 DNA polymerase and the mitochondrial DNA polymerase gamma demonstrate tight DNA binding in the presence of their respective processivity factors (Table S1). However, for apPol, no processivity factor has been identified to date, raising the possibility that apPol might have an intrinsically high affinity for DNA, allowing the enzyme to perform replicative synthesis without a processivity factor as is seen for the bacteriophage phi29 DNA polymerase.^22^

We performed primer extension assays under burst conditions with varying concentrations of substrate S1 to determine apPol’s affinity for DNA. During the fast phase of product formation, only the fraction of DNA that preforms the binary complex (Figure 1B, step 2) gets extended. Therefore, the amplitude of the fast phase is indicative of the concentration of the productive preformed binary complex. Thus, by monitoring the amplitudes of the fast phase, we determined the apparent affinity of apPol for DNA (K_Dapp_,^DNA^) and calculated the active fraction of the apPol (Figure S1A,B) as 104 ± 6.4 nM, indicating that ∼22% of the enzyme was active in our preparation. We note that this is a slight underestimation of the active apPol concentration.^23^ Using this active fraction, we performed global fitting of all product formation time courses to determine the rate constants governing the minimal kinetic pathway for correct dNTP incorporation by apPol.

The sequence of events for the minimal pathway (Figure 2B) is similar to the general kinetic scheme (Figure 1B) with the following exceptions. We assumed PP_i_ release to be fast and irreversible such that this step was not explicitly modeled. We could not detect pyrophosphorolysis, that is, the reverse of the phosphodiester bond formation, in the presence of 800 nM PP_i_ (the highest concentration of PP_i_ that could be generated in our single nucleotide primer extension assays) (Figure S1C) which supported the assumption of PP_i_ release being essentially unidirectional under our reaction conditions. With rapid PPi release, no build-up of the postchemistry ternary complex (Figure 1B, Pol·DNA_*n*+1_·PPi) should occur, and it may be anticipated that any slow reversibility of the chemistry step will not become apparent under our experimental conditions. Therefore, we modeled bond formation to be irreversible (Figure 2B, step3). We emphasize that the kinetic pathway used for global fitting is perhaps the minimal catalytic cycle of apPol. As has been found with other DNA polymerases,^24,25^ we expect one or more conformational steps to lead the ground-state prechemistry ternary complex (Figure 2A, Pol·S1_*n*_·dTTP) to a catalytically competent state. However, we have not modeled these steps explicitly since the kinetic experiments performed in this study would not be able to constrain the rate constants governing the conformational changes. The kinetic constants governing our minimal model were determined by numerical integration and readily explained all the presteady-state experimental data (Figure 2C–E) with 1D and 2D confidence contour analysis indicating that all rate constants are well-constrained (Figure S2).

Based on global fitting, we determined the dissociation constant (*K*_D_^DNA^) for the prechemistry binary complex to be 77.9 nM (Figure 2B; *k*_–1_/*k*_1_, 2C). Compared to other replicative DNA polymerases, apPol has a somewhat weaker affinity for DNA (Table S1). The weak *K*_D_^DNA^ can result from a rapid dissociation of the binary complex or a slow association of apPol and DNA. To ascertain the cause of the weak *K*_D_^DNA^, we directly measured the dissociation rate of the apPol·DNA binary complex from a double-mixing experiment (Figure 2D, inset). We preincubated apPol with fluorescently labeled DNA substrate S1 to form the apPol·S1 binary complex. This sample was then incubated with an excess unlabeled DNA trap for varying time intervals. Any polymerase molecule that dissociated from the labeled DNA would bind to the unlabeled trap. Thus, with increasing incubation time, the concentration of the apPol·S1 complex would decrease as a function of the dissociation rate of the binary complex. After incubation, a saturating amount of dTTP was added to extend S1 still bound to apPol by a single nucleotide, and the reaction was terminated after 170 ms. The concentration of the extended labeled DNA product was plotted as a function of the first incubation time (Figure 2D), and the data were globally fit to the minimal pathway. The dissociation rate constant (*k*_–1_) was 1.9 s^–1^ (Figures 2B and S2C; *k*_–1_), which is 1 to 2 orders of magnitude faster than the k_–1_ for most replicative DNA polymerases in the presence of their corresponding processivity factors (Table S1). The association rate constant for the apPol·DNA binary complex (k_1_) was 24.4 μM^–1^ s^–1^ (Figures 2B and S2C; *k*_1_), indicating a diffusion-limited association of the binary complex. Taken together, our results indicate that apPol has a weak affinity for its DNA substrate primarily due to the rapid dissociation of the apPol·DNA binary complex.

### apPol Incorporates Nucleotides with Low Efficiency and Processivity

During genome duplication, a high efficiency of dNTP incorporation, combined with high processivity, ensures that replicative DNA polymerases can rapidly add tens of thousands of nucleotides to the growing DNA strand. The efficiency of nucleotide incorporation is determined by two kinetic parameters, the affinity of the nucleotide for the binary complex (*K*_D_^dNTP^) and the rate of dNTP incorporation. In order to determine these two parameters, we performed primer extension assays under burst conditions using DNA substrate S1 and varied the dTTP concentration (Figure 2E). *k*_fast_ increased with the increasing concentration of dTTP and saturated hyperbolically as a function of the incoming nucleotide concentration (Figure S1D,E). This indicates that nucleotide binding to the prechemistry binary complex rapidly equilibrates and is followed by a slower catalytic step. From global fitting, the dissociation constant for the apPol·DNA·dNTP prechemistry ternary complex (*K*_D_^dNTP^) was determined to be 131 μM (Figures 2B and S2C; k_–2_/10 μM^–1^ s^–1^), and the rate constant governing nucleotide addition was 30 s^–1^ (Figures 2B and S2C; *k*_3_). Compared to other replicative polymerases, *K*_D_^dNTP^ of apPol was weaker by almost 1 or 2 orders of magnitude (Table S1). For instance, the mitochondrial DNA polymerase gamma holoenzyme (Pol gamma) has a *K*_D_^dNTP^ of 0.78 μM, while the *K*_D_^dNTP^ for mammalian DNA polymerase delta-PCNA complex (Pol delta-PCNA) is 1 μM (Table S1). The weak *K*_D_^dNTP^ of apPol, combined with a modest rate of bond formation, resulted in a low efficiency (*k*_3_/*K*_D_^dNTP^) of correct nucleotide incorporation (0.23 μM^–1^s^–1^) (Table S1).

We calculated the ratio of the rate constants governing the steps of bond formation and DNA dissociation (k_3_/k_-1_) for apPol to be ∼16 (Figure 2B, Table S1)—2 to 3 orders of magnitude lower compared to the corresponding ratio for typical replicative DNA polymerases in the presence of their corresponding processivity factors (Table S1). Taken together, we show that under our experimental conditions, apPol performs nucleotide incorporation with low efficiency and processivity when compared to most other replicative DNA polymerases. This raises the possibility that the interaction of apPol with other components of the apicoplast replisome might influence these kinetic parameters and convert apPol into an efficient replicative DNA polymerase.

**Figure 3:**
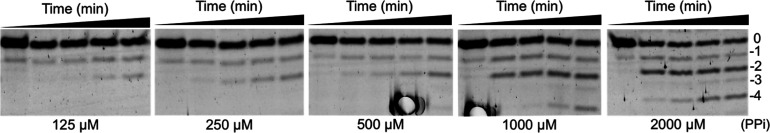
Pyrophosphorolysis catalyzed by apPol. The five panels depict 15% acrylamide-urea denaturing gels showing primer degradation in the presence of increasing (from left to right) concentrations of inorganic pyrophosphate (PP_i_). A final concentration of 104 nM active apPol was incubated with 100 nM of DNA substrate S1 and varying concentrations of PP_i_ ranging from 125 to 2000 μM in apPol reaction buffer. Reactions were incubated at 37°C for various time intervals (0, 0.33, 0.5, 1, and 2 min) before quenching with excess EDTA. 0: the starting primer without any degradation. −1, −2, −3, and −4: primer strand degraded by one, two, three, and four nucleotides, respectively.

Rapid enzyme turnover is a necessity for efficient catalysis. Therefore, as a general rule, the product(s) of an enzyme-catalyzed reaction has(have) a very low affinity for the enzyme itself. Consistent with this, when DNA polymerases catalyze nucleotide incorporation, the PP_i_ byproduct typically gets released rapidly from the postchemistry ternary complex (Figure 1B, step 4), and this step generally occurs prior to or in conjunction with the translocation step of the polymerization cycle,^26−28^ ensuring that bond formation (Figure 1B, step 3) is essentially irreversible. The elongated DNA (Figure 1B, DNA_n+1_) is an exception to the general rule of fast product release because the product DNA from one round of nucleotide incorporation becomes the substrate for the next round, and hence, the extended DNA typically has a high affinity for the DNA polymerase. In vitro*,* the build-up of a high concentration of PP_i_ in the presence of a DNA polymerase has been shown to result in pyrophosphorolysis, that is, the reversal of the phosphodiester bond formation.^25^ We performed primer degradation assays in the presence of varying concentrations of PP_i_, and primer degradation could be detected with 250 μM PP_i_ with the degradation increasing at higher PP_i_ concentrations, indicating that apPol can catalyze robust pyrophosphorolysis (Figure 3).

Based on the lack of product formation in primer extension assays performed with a nucleotide analogue where the bridging oxygen between the β and γ phosphates was replaced by a nitrogen, it has been proposed that the PP_i_ generated during apPol-mediated dNTP incorporation gets hydrolyzed to inorganic phosphates (P_i_), followed by dissociation of the P_i_ from the postchemistry complex.^29^ This hypothesis is in contradiction to our observation. If it was necessary for PP_i_ to be hydrolyzed to P_i_ before product release from the apPol postchemistry complex, then P_i_, and not PP_i_, could bind to the apPol·DNA binary complex. Consequently, PP_i_ would not be able to drive apPol-catalyzed pyrophosphorolysis. However, as mentioned above, apPol performs robust pyrophosphorolysis in the presence of PP_i_. Moreover, our attempts to measure the amount of P_i_ released during primer extension by apPol could not detect any P_i_ above the background (Figure S1F). While we conclude that apPol does not hydrolyze PP_i_ to P_i_, it remains possible that such hydrolysis is required for the functioning of certain TLS polymerases as has been reported previously.^29,30^

### apPol Translocates Rapidly along the DNA Substrate

**Figure 4:**
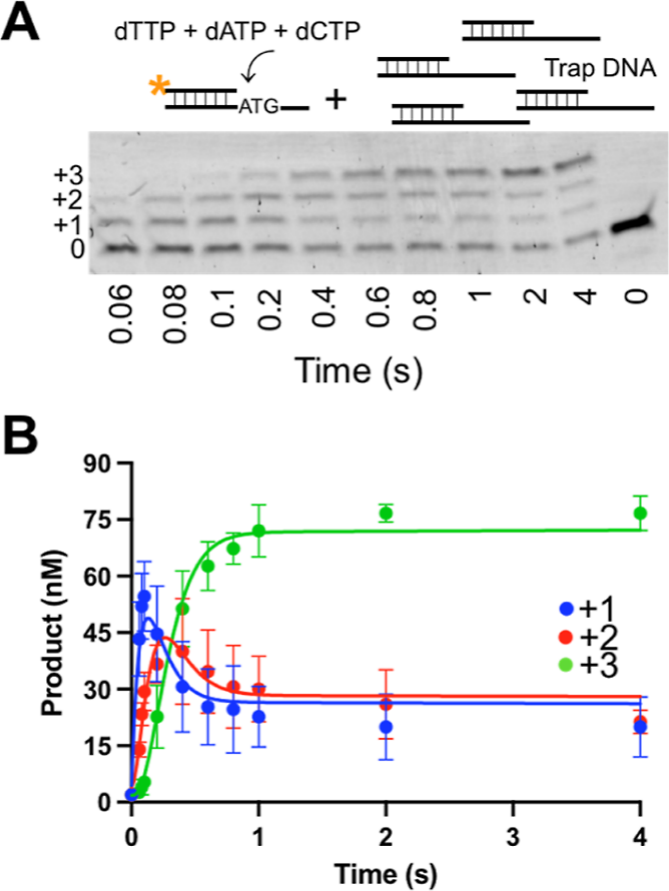
Kinetics of processive synthesis by apPol. (A) Representative acrylamide-urea gel showing primer extension in the presence of multiple nucleotides. The schematic of the reactants (substrate S1, the nucleotides, and the trap DNA) added is shown above the gel. A final concentration of 200 nM DNA substrate S1 was incubated with 104 nM active apPol; 250 μM each of dTTP, dATP, and dCTP; and 50 μM trap DNA and incubated together at 37°C for various time intervals ranging from 0 to 4 s. The reactions were quenched with 250 mM EDTA and analyzed on an acrylamide-urea gel. *, orange: FAM label. 0: unextended primer. +1, +2, and +3: primer strand extended by one, two, and three nucleotides, respectively. (B) Concentrations of the DNA primer strands extended by one (blue), two (red), and three (green) nucleotides from (A) plotted as a function of reaction time. The smooth lines overlaying the datapoints represent the best global fit of the data to the kinetic scheme shown in Figure S3A. The experiment was performed in triplicate, and the average of the three independent data sets is plotted in (B). Error bars represent SD.

In vivo*,* replicative polymerases perform processive synthesis by translocating along the DNA substrate and moving the next base on the template strand (Figure 1C, T_1_) to the templating position (Figure 1C, T_0_). In this scenario, the slow, often rate-limiting step of postchemistry binary complex dissociation (Figure 1B, step5) does not occur after every dNTP addition and is instead replaced by the translocation step (Figure 1B; “translocation”). The rate of translocation has been estimated directly or indirectly for several DNA polymerases and is typically a fast process.^31,32^ On the other hand, a recent kinetic study found PP_i_ release and translocation to be partially rate-limiting for the T7 DNA polymerase–thioredoxin complex (T7 Pol) at low temperatures.^33^ To assess apPol’s speed of translocation, we mimicked processive synthesis by performing a primer extension assay with substrate S1 in the presence of multiple nucleotides. We included 250 μM each of dTTP, dATP, and dCTP along with an excess of the unlabeled DNA trap (Figure 4A). The templating sequence of S1 (Figures 1C and 4A) is such that the addition of these three dNTPs should result in the elongation of the primer strand by exactly three nucleotides. The presence of trap DNA ensured that apPol molecules that dissociate from the fluorescently labeled DNA are sequestered and cannot rebind labeled S1. The three extension products (Figure 4A) were quantitated and graphed individually as a function of time (Figure 4B). Our data could be explained by a simplified mechanism mimicking processive synthesis (Figure S3A), where fast translocation is assumed, and therefore, this step was not explicitly modeled. The average rate of nucleotide addition was 8.4 s^–1^—a rate that is comparable to the k_fast_ of 18.8 s^–1^ recorded from the single nucleotide incorporation experiment in the presence of 250 μM dTTP (Figure S1D,E), with the rates of all three individual nucleotide additions being similar (Figure S3D). Taken together, our results indicate that the rate of translocation is at least comparable to the rate of bond formation and is not rate-limiting during processive synthesis by apPol.

### Kinetics of Lesion Bypass by apPol

The oxidizing environment of the apicoplast indicates that apDNA and the apicoplast nucleotide pool will encounter high levels of reactive oxygen species (ROS).^34^ ROS are potent mutagens and cause oxidative damage to nucleobases.^35^ As the only DNA polymerase within the apicoplast, we anticipate apPol to encounter ODNs. Using presteady-state primer extension assays, we examined whether apPol can perform efficient TLS on encountering an ODN in the template strand.

### apPol Can Bypass ODNs

**Figure 5:**
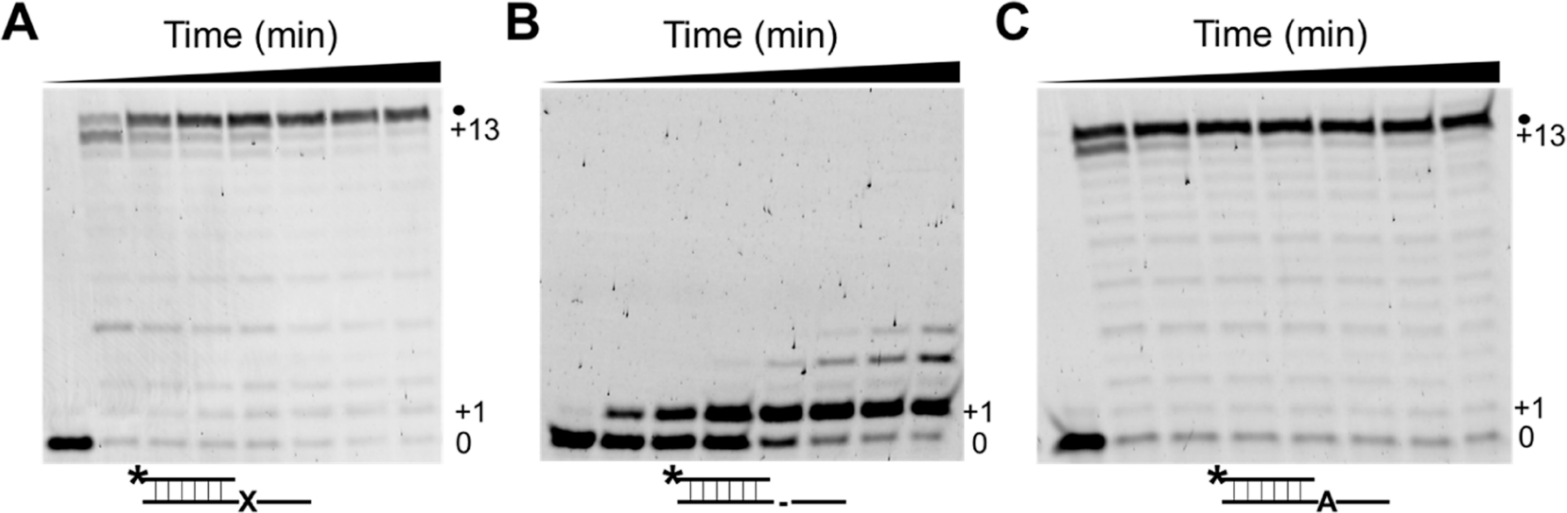
Translesion synthesis by apPol in the presence of multiple nucleotides. (A–C) Acrylamide-urea gels depicting multiple nucleotide extensions of DNA substrate S2 (8-oxo-dGMP at the templating position) (A), S3 (abasic site at the templating position) (B), and S4 (undamaged DNA) (C). The schematics of the DNA substrates are shown below the respective gels. In all three cases, a final concentration of 104 nM active apPol was incubated with 200 nM DNA and 250 μM of each of the four dNTPs. The reactions were incubated at 37°C for various time intervals ranging from 0 to 20 min (0, 0.5, 1, 2, 5, 10, 15, and 20 min) and then quenched by adding excess EDTA. The reactions were analyzed on a 15% acrylamide-urea gel. 0: 23 nucleotide long primer strand (this is the starting length of the primer), +1: primer strand extended by one nucleotide, +13: primer strand extended by 13 nucleotides, which is the length of the 5′ overhang of the template strand, and •: 37 nucleotides’ long primer stand. The length is one nucleotide more than the length of the template strand and is formed due to one nontemplated nucleotide addition by apPol. *: the FAM label on the primer strand, X: 8-oxo-dGMP, -: abasic site, and A: dAMP.

We first investigated whether apPol can replicate over a templating ODN and performed multiple nucleotide incorporation assays with ODN-containing DNA substrates (Figure 1C). We tested two critical lesions: 8-oxo-7,8-dihydroguanosine monophosphate (8-oxo-dGMP) and apurinic/apyrimidinic site (abasic site) (Figure 1C, substrates S2 and S3, respectively) and found that apPol can bypass both the ODNs, albeit to different extents (Figure 5). apPol performed a robust bypass of 8-oxo-dGMP and could extend till the end of the DNA template (Figure 5A). However, the bypass of the abasic site was considerably less efficient with extension past the lesion being negligible (Figure 5B). Moreover, we noticed that upon reaching the end of the DNA, apPol added an extra nucleotide to the nascent DNA strand (Figure 5A,C, “•”), and this nontemplated nucleotide addition was independent of the presence of an ODN.

### apPol Preferentially Adds dATP opposite an Abasic Site, but the Bypass Is Inefficient

**Figure 6:**
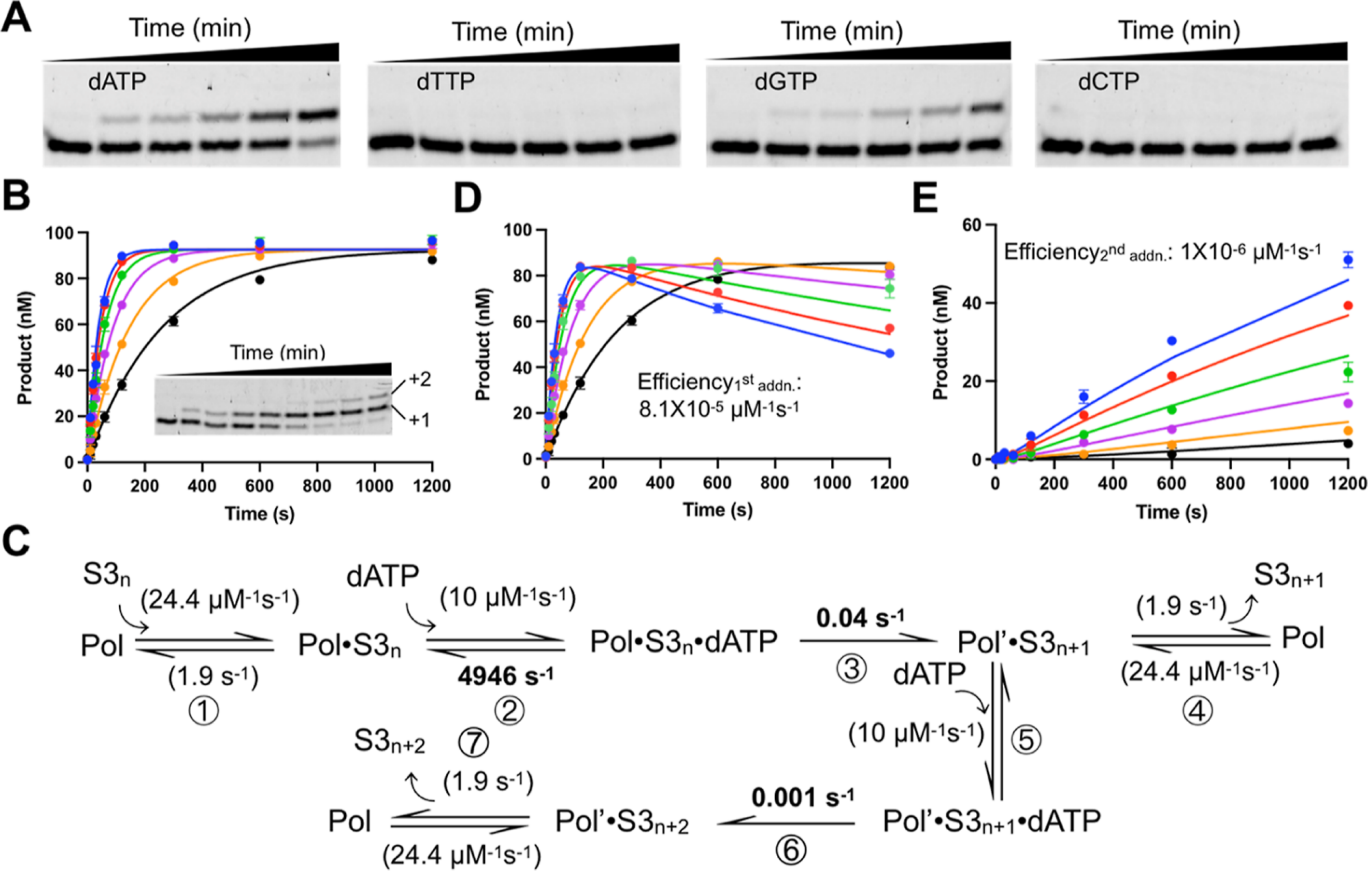
Kinetics of abasic site bypass by apPol. (A) Acrylamide-urea gels depicting apPol-mediated single nucleotide incorporations in DNA substrate S3. Reactions were set up at 37°C with a final concentration of 104 nM active apPol, 100 nM substrate S3, and 250 μM of incoming dNTP (from left: dATP, dTTP, dGTP, and dCTP) and incubated for varying time intervals (0, 0.33, 0.5, 1, 2, and 5 min) following which an excess of EDTA was added to quench the reactions before being analyzed on 15% acrylamide-urea gels. (B) Global fitting of the time courses from primer extension assays to determine the efficiencies of dATP incorporation by apPol opposite an abasic site and extension from the dAMP:abasic site basepair. **Inset**: Acrylamide-urea gel depicting the time course of primer extension with 500 μM dATP. +1 and +2: primer strand extended by one and two nucleotides, respectively. Single nucleotide primer extension assays were performed as described for (A) with the following changes. The incoming nucleotide was dATP for all the assays, and the concentration of the nucleotide was varied from 62.5 μM to 2 mM (62.5 μM: black, 125 μM: orange, 250 μM: magenta, 500 μM: green, 1 mM: red, and 2 mM: blue), and the time interval for incubation ranged from 0 to 20 min (0, 0.17, 0.33, 0.5, 1, 2, 5, 10, and 20 min). Due to the sequence of the T_0_ and T_1_ nucleobases in substrate S3, we could detect two dATP addition events. The time courses show the combined product concentrations (i.e., the total of the +1 and +2 products). The smooth lines overlaying the datapoints are the best global fit of the data. (C) Kinetic pathway used for global fitting of dATP incorporation opposite the abasic site and extension past the lesion. The best fit rate constants are shown next to the corresponding steps. Rate constants shown in parenthesis were held constant during global fitting. K_-5_ and k_6_ were not individually constrained by the data; however, their ratio was well-constrained; therefore, only k_6_ is shown. S3_*n*_: Substrate S3 with 23 nucleotide long unextended primer strand. S3_*n*+1_ and S3_*n*+2_: S3 with a primer strand extended by one and two nucleotides, respectively. Pol′: apPol translocated by one nucleotide. (D,E) Global fitting of the +1 (D) and +2 (E) products of (B) using model shown in (C). Experiments were performed in triplicate. Error bars represent SD.

Abasic sites are particularly detrimental as they present a unique challenge to polymerases due to the complete loss of base information from the templating strand. To determine which nucleotide is incorporated by apPol opposite the abasic site, we performed single nucleotide incorporation assays using DNA substrate S3 (Figure 1C) while varying the identity of the incoming nucleotide. We found that apPol preferentially adds dATP (Figure 6A) opposite an abasic site but can also add dGTP at a much slower rate. dATP addition can either occur by the so-called “A-rule”,^36^ a mechanism shared by other A-family DNA polymerases for abasic site bypass^37,38^ or, owing to the templating sequence in S3, could occur through dNTP-stabilized misalignment.^39^ apPol was unable to incorporate any of the pyrimidines in this context (Figure 6A).

In order to understand the relevance of abasic site bypass by apPol, we determined the efficiency of dATP incorporation opposite this lesion. We performed primer extension assays with substrate S3 and varied the concentration of dATP (Figures 6B and S4A). Although the experiments were performed under burst conditions, we could not detect any burst of product formation. This would indicate that for abasic site bypass, apPol performs distributive synthesis such that the postchemistry steps were not rate-limiting. The rate of incorporation saturated hyperbolically as a function of dATP concentration (Figure S4B), indicating that nucleotide binding to the binary complex is in rapid equilibrium, followed by chemistry. We fit the primer extension data using the KinTek Explorer (Figure 6B–E, S5A-C) and found that the efficiency of dATP incorporation opposite an abasic site lesion (8.1 × 10^–5^ μM^–1^ s^–1^; Figure 6C,D, k_3_k_2_/k_-2_) is more than 3 orders of magnitude worse than the efficiency of dNTP incorporation opposite an undamaged nucleotide (0.23 μM^–1^s^–1^, Table S1). The low efficiency results primarily from a 750-fold slower rate of bond formation opposite the abasic site, with a rate constant of 0.04 s^–1^ (compare *k*_3_ from Figures 6C and 2B). In contrast, apPol’s affinity for nucleotide (*K*_D_^dATP:abasic^) is only 4-fold weaker (524 μM; Figure 6C) than the corresponding *K*_D_^dNTP^ for undamaged DNA (131 μM, Table S1). Taken together, these results indicate that, by itself, apPol can only perform inefficient, distributive synthesis while bypassing an abasic site.

In substrate S3 (Figure 1C), the T_1_ base after the abasic site is a thymine, resulting in the possibility of a second A being incorporated after bypassing the abasic site lesion. Indeed, while performing primer extension assays with dATP, we observed two nucleotide incorporations at longer incubation times (Figure 6B inset)—dATP incorporation opposite the abasic site (+1) followed by dATP incorporation opposite the dTMP at position T_1_ (+2). Having quantitated the two extension products separately, we could readily fit the data to a kinetic model with two bond formation steps (Figure 6C). While the accurate determination of the *K*_D_^dNTP^ and rate of incorporation for the second nucleotide could not be made, we could estimate the efficiency of dATP addition opposite the T_1_ base (Figures 6D,E and S5A–C) and found that incorporation past the abasic site was extremely inefficient (∼1 × 10^–6^ μM^–1^ s^–1^), over 70-fold worse than the incorporation efficiency opposite the abasic site itself, hinting that apPol-mediated bypass is not the major route for negotiating abasic sites in the apicoplast.

### apPol Performs Distributive Error-Prone Synthesis opposite 8-Oxo-dGMP

**Figure 7:**
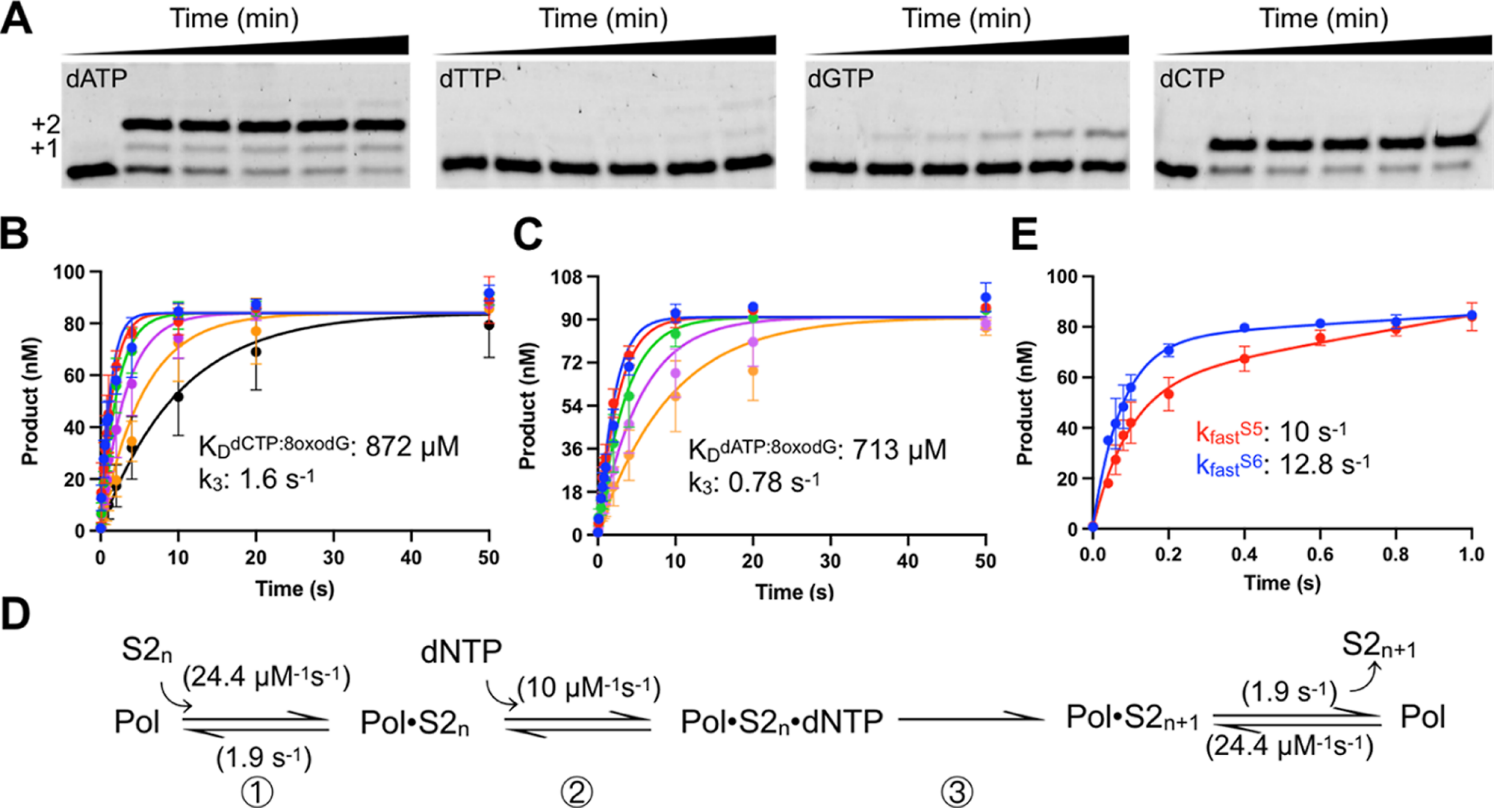
Kinetics of 8-oxo-dGMP bypass by apPol. (A) Acrylamide-urea gels depicting apPol-mediated single nucleotide incorporations to DNA substrate S2 in the presence of (from left) dATP, dTTP, dGTP, and dCTP. +1 and +2: primer strand extended by one and two nucleotides, respectively. Single nucleotide primer extension assays were set up at 37°C with a final concentration of 104 nM active apPol, 100 nM DNA substrate S2, and 250 μM of incoming dNTP. The reactions were incubated for varying time intervals ranging from 0 to 5 min (0, 0.33, 0.5, 1, 2, and 5 min) following which an excess of EDTA was added to quench the reactions, and the samples were analyzed on 15% acrylamide-urea gels. (B,C) Global fitting of the time courses from primer extension assays to determine the efficiency of dCTP or dATP incorporation by apPol opposite 8-oxo-dGMP. Single nucleotide primer extension assays were performed as described for (A) with the following changes. The incoming nucleotide was either dCTP (B) or dATP (C), and the concentration of the nucleotide was varied from 62.5 μM to 2 mM for dCTP addition (62.5 μM: black, 125 μM: orange, 250 μM: magenta, 500 μM: green, 1 mM: red, and 2 mM: blue) and 125 μM to 2 mM for dATP addition (125 μM: orange, 250 μM: magenta, 500 μM: green, 1 mM: red, and 2 mM: blue), and the maximum incubation time was 50 s. Due to the sequence of the T_0_ and T_1_ nucleobases in substrate S2, we could detect two dATP addition events. The time courses in (C) show the combined product concentrations (i.e., the total of the +1 and +2 products). The smooth lines overlaying the datapoints are the best global fit of the data. The rate constant governing dCTP or dATP addition (k_3_) and the affinity of the nucleotide for the binary complex (*K*_D_^dNTP:8oxodG^, where dNTP is either dCTP or dATP) are shown as insets with the corresponding graphs. (D) Kinetic pathway used for global fitting of the primer extension assays shown in (B,C). Rate constants shown in parenthesis were not allowed to float during global fitting. S2_*n*_: Substrate S2 with 23 nucleotide long unextended primer strand. S2_n+1_: Substrate S2 with the primer strand extended by one nucleotide. (E) Time courses from primer extension assays performed with DNA substrates S5 (red) and S6 (blue). Single nucleotide primer extension assays were performed as described for (A) with the following changes. The incoming nucleotide was dATP (final concentration 2 mM), and the maximum incubation time was 1 s. The time courses were fit to the full burst equation, and the rates of the fast phases are mentioned in the inset. All experiments were performed in triplicate, and the average of the three independent data sets is plotted in the graphs shown in (B,C,E), while the error bars represent the SD of data sets.

8-oxo-dG is the most abundant of the oxidative lesions. A large number of DNA polymerases, including several A-family polymerases, can incorporate either dCTP or dATP opposite this lesion, albeit with varying degrees of efficiency and fidelity.^40,41^ The promiscuity arises from the ability of 8-oxo-dGMP to efficiently pair with either dCTP (Watson–Crick pairing) or dATP (Hoogsteen pairing).^42^ From single nucleotide primer extension assays with DNA substrate S2 (Figure 1C), we found that apPol could incorporate both dCTP and dATP opposite 8-oxo-dGMP (Figure 7A). We could not detect any dTTP addition, while dGTP was added very slowly (Figure 7A).

To determine how efficiently apPol incorporates dCTP or dATP opposite 8-oxo-dGMP, we performed single nucleotide primer extension assays with varying concentrations of incoming dCTP or dATP (Figures 7B,C and S4C,E). Although both sets of assays were set up under presteady-state burst conditions, no “burst” of product formation could be detected. This indicates that unlike nucleotide incorporation opposite an undamaged template, chemistry (or a step preceding chemistry) is rate-limiting in apPol’s catalytic cycle of nucleotide incorporation opposite 8-oxo-dG. For both dCTP and dATP, increasing the concentration of the incoming nucleotide increased the rate of primer extension (*k*_obs_), and the rate saturated hyperbolically as a function of the dNTP concentration (Figures S4D,F). Consequently, we performed global fitting of the primer extension data to a kinetic scheme characterized by the rapid equilibrium of dNTP binding, followed by slow chemistry (Figure 7D). Based on this fit, we found that apPol inserts dCTP and dATP with almost equal efficiencies (k_3_/K_D_^dNTP:8oxodG^) of 1.8 × 10^–3^ μM^–1^s^–1^ and 1.1 × 10^–3^ μM^–1^s^–1^, respectively (Figures 7B,C and S6C,F and Table S2). This indicates that apPol does not strongly discriminate between correct and incorrect incorporations opposite 8-oxo-dG. However, these efficiencies were 2 orders of magnitude less than the efficiency of correct incorporation opposite an undamaged template, suggesting that apPol can sense the damaged templating base. The lower efficiency was the combined effect of a nearly 30-fold reduced rate of incorporation (1.6 and 0.78 s^–1^ for dCTP and dATP, respectively, compared to 30 s^–1^ for dTTP incorporation opposite dAMP) and a 7-fold weaker dNTP binding (872 and 713 μM for dCTP and dATP incorporation opposite 8-oxo-dGMP, respectively, compared to 131 μM for dTTP incorporation opposite dAMP) (Tables S1 and S2).

TLS consists of two parts: incorporation of a nucleotide opposite the lesion, followed by extension past the lesion. To investigate the kinetics of extension past 8-oxo-dGMP, we used substrates S5 and S6 (Figure 1C). S5 has dCMP already added to the primer strand opposite 8-oxo-dGMP, while S6 has dAMP in place of the dCMP. We monitored the kinetics of dATP incorporation by performing primer extension under the presteady-state burst condition. In both cases, the time courses were biphasic, with *k*_fast_ of ≥10 s^–1^ (Figure 7E), over 5 times faster than the rate of DNA dissociation from the postchemistry binary complex (Figure 2B), suggesting that apPol resumes processive synthesis immediately after incorporation opposite 8-oxo-dGMP. We attempted to determine the efficiency of extension by varying the dATP concentration; however, our preliminary experiments indicate that the affinities of dATP for apPol·S5 and apPol·S6 binary complexes are very weak (data not shown) and prevent accurate determination of efficiencies.

### apPol’s Proofreading Activity Does Not Discriminate between Accurate and Error-Prone Bypass of 8-Oxo-dGMP

**Figure 8:**
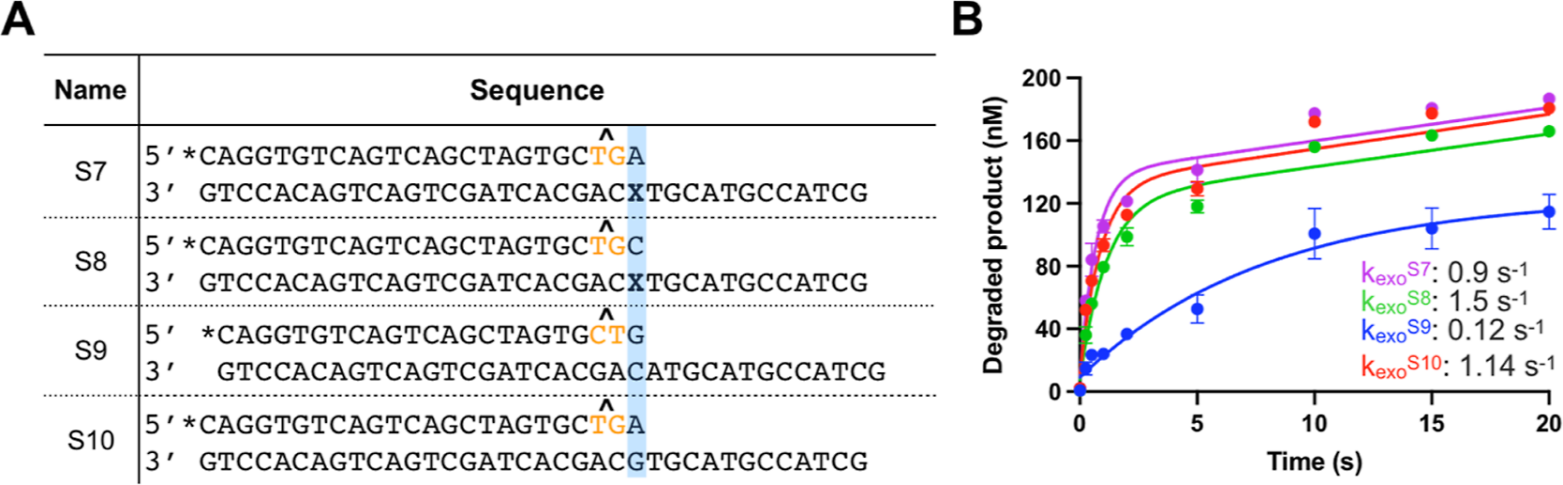
Proofreading exonuclease activity of apPol^WT^. (A) DNA substrates were used to analyze the proofreading activity of apPol^WT^. Bases in the primer strand at positions P_–2_ and P_–3_ are highlighted in orange, while the basepairs probed in the exonuclease assays are shaded blue. The oxidatively damaged nucleotides are in bold. *: FAM label, **X**: 8-oxo-7,8-dihydroguanosine monophosphate (8-oxo-dGMP), and **^**: phosphorothioate linkage. (B) Primer degradation assays for apPol^WT^. The concentration of the primer strand shortened by a single nucleotide (degraded products) is plotted as a function of time. A final concentration of 104 nM active apPol^WT^ was incubated with 200 nM of DNA substrate (S7 magenta, S8 green, S9 blue, or S10 red), and the reaction was initiated by adding a final concentration of 10 mM Mg^2+^. Reactions were incubated at 37°C for various time intervals (0, 0.25, 0.5, 1, 2, 5, 10, 15, and 20 s) and then quenched with excess EDTA. Samples were analyzed on a 15% acrylamide-urea denaturing gel, and the concentration of degraded product was plotted as a function of time. The data were fit to the burst equation, and the rate of the fast phase was approximated as the rate of the exonucleolytic cleavage (*k*_exo_). *k*_exo_ for S7, S8, S9, and S10 were 0.9 ± 0.1, 1.5 ± 0.2, 0.12 ± 0.05, and 1.14 ± 0.2 s^–1^, respectively. All experiments were performed in triplicate, the average of the three independent data sets was plotted, and the error bars represent the SD of data sets.

The 3′ to 5′ exonuclease proofreading activity of apPol might act as a checkpoint to discriminate between correct and incorrect incorporation opposite 8-oxo-dGMP by efficiently excising out the dAMP mispaired with 8-oxo-dGMP. We investigated if this was indeed the case by performing apPol-catalyzed exonuclease assays with DNA substrates S7–S10 (Figure 8). For these experiments, we used wild-type apPol (apPol^WT^) lacking the D82N and E84Q point mutations, and all DNA substrates had a phosphorothioate linkage between the P_-2_ and P_-3_ positions of the primer (Figure 8A), which limited any exonucleolytic degradation beyond a single nucleotide.

The exonuclease assays were performed under the presteady-state burst condition, and we detected a biphasic time course of primer degradation indicating the presence of a slow step following the exonucleolytic cleavage. On comparing the rates of the fast phase of the primer degradation time courses, we found that DNA substrates containing a dAMP:8-oxo-dGMP or dCMP:8-oxo-dGMP pair at the 3′ end of the primer (Figure 8A, substrates S7 and S8, respectively) were cleaved at comparable rates of 0.9 ± 0.1 and 1.5 ± 0.2 s^–1^, respectively (Figure 8B, magenta and green time courses, respectively), indicating that the proofreading domain of apPol^WT^ does not discriminate between correct and incorrect pairing opposite 8-oxo-dGMP. Since the rate of extension past the 8-oxo-dGMP lesion is > 10-fold faster than the rate of excision (comparing k_obs_, Figure 7E with k_exo_, Figure 8B), we can predict a high probability of a dAMP:8-oxo-dGMP mispair becoming integrated into the apDNA.

We also measured the rates of cleavage of undamaged DNA substrates (Figures 8A and S9 and S10) by apPol^WT^. In substrate S9, the primer and template strands are complementary to each other, while in S10, there is a mispair at the 3′ end of the primer. The primer degradation rate for S9 was almost 10-fold lower than the corresponding rate for S10 (0.12 ± 0.05 s^–1^ for S9 and 1.14 ± 0.2 s^–1^ for S10; Figure 8B), indicating that the proofreading active site of apPol^WT^ can discriminate between correctly paired and mispaired DNA substrates. Interestingly, the exonuclease rates for substrates S10 (mispaired undamaged DNA) and S8 (dCMP:8-oxo-dGMP at the 3′ end of the primer strand) are comparable and >10-fold higher than the exonuclease rate for S9 (perfectly paired undamaged DNA), suggesting that the dCMP:8-oxo-dGMP pair does not adopt a conventional Watson–Crick base pair geometry and is recognized as a mispair by apPol^WT^.

## Discussion

In this work we present the first comprehensive kinetic analysis of the *P. falciparum* apicoplast DNA polymerase (apPol) and investigate the lesion bypass activity of this enzyme. The implications of our results in the context of apicoplast genome duplication are summarized below.

### Kinetics of apPol-Mediated DNA Synthesis; Implication for Apicoplast Genome Duplication

Compared to other replicative DNA polymerases, apPol has surprisingly low efficiency and processivity, more in line with those of DNA polymerases involved in DNA repair and TLS (Table S1). Moreover, owing to its low affinity for dNTP, apPol’s processivity inside the apicoplast might be further lowered unless the apicoplast dNTP pool concentration is significantly higher than the known concentrations of bacterial, eukaryotic, or mitochondrial nucleotide pools.^43−45^ Therefore, if apPol copies the apicoplast genome on its own, then it must do so using a nonprocessive mechanism, unlike other replicative polymerases. As a part of the replisome, a DNA polymerase interacts with various components of the replication machinery. Nearly all replicative polymerases interact with a partner protein called the processivity factor,^46^ which increases the processivity of a polymerase either by reducing the dissociation rate of the prechemistry binary complex or by increasing the rate of chemistry.^16,47^ In certain cases, a processivity factor may even increase the polymerase’s affinity for nucleotides.^48^ However, to date, no processivity factor for apPol has been identified, and it has been proposed that the relatively small size of the apicoplast genome (∼35 kb) might allow apPol to perform replicative synthesis without an associated factor.^5^ It remains to be seen if components such as the helicase or SSB of the apicoplast replisome or an as-yet-undiscovered protein influence apPol’s efficiency and processivity. Alternatively, it is possible that the bulk of the apicoplast genome is copied by a DNA polymerase that has not been identified yet. Future studies looking at the activity of apPol as part of the apicoplast replisome will shed more light on our understanding of how this polymerase duplicates the apicoplast DNA.

### Translesion Synthesis by apPol Might Be a Source of Mutation for the Apicoplast Genome

Inside a human host, apicoplast replication occurs during the blood stage of *Plasmodium’s* life cycle.^5^ In addition to the high levels of ROS in erythrocytes, *Plasmodium* infection leads to further ROS production as part of the host’s defense mechanism.^49^ Moreover, many conventional antimalarials like quinolones utilize a ROS-mediated mechanism to kill *Plasmodium*.^50^ ROS are a potent mutagen that generates ODNs.^35^ The restrictive active sites of replicative polymerases prevent efficient nucleotide incorporation against damaged nucleotides, which, in turn, can promote replication fork collapse triggering cell death. One of the strategies used to prevent this catastrophe is to deploy specialized error-prone TLS polymerases to the stalled fork. While these polymerases can bypass the lesion, they may introduce mutations during the bypass. In its role as the only known DNA polymerase within the apicoplast, we expect apPol to encounter and effectively copy oxidatively damaged DNA.

We found that apPol can bypass both 8-oxo-dGMP and abasic site lesions with varying efficiencies. 8-oxo-dGMP is the most abundant of the ODNs and can be present in the DNA through in situ oxidation of dGMP or due to incorporation of the lesion by a DNA polymerase.^35^ Abasic sites are formed as a result of hydrolysis of the base moiety of a nucleotide and are arguably the most deleterious of the ODNs.^51^ In addition to ROS-mediated generation, abasic sites can be formed via a variety of different routes including spontaneous hydrolysis of the DNA.^52^ We show that, unlike the robust bypass of 8-oxo-dGMP, incorporation of a nucleotide opposite the abasic site was much slower, with ∼3000-fold lower efficiency of bypass (8.1 × 10^–5^ μM^–1^ s^–1^) compared to the efficiency of incorporation opposite an undamaged DNA (Table S1). Our data indicate that in the apicoplast, apPol might not bypass the majority of the abasic sites and that a separate pathway is probably used to handle this lesion. Similar to apPol, Pol gamma is inefficient at bypassing abasic sites, and in the mitochondria, the base excision repair (BER) pathway repairs the majority of abasic site lesions and prevents mitochondrial DNA degradation.^53^ An intact long patch BER pathway has been identified in apicoplast,^5^ and it is possible that this pathway is the primary route for abasic site removal. Unlike apPol and Pol gamma, the A-family chloroplast DNA polymerases can bypass and extend past an abasic site with high efficiency,^54^ underscoring the inherent differences in TLS mechanisms among the organellar replicative polymerases.

In contrast to helix-distorting lesions like an abasic site, 8-oxo-dG do not block replication. Rather, this lesion is a potent mutagen.^55^ If left unrepaired, an 8-oxo-dGMP: dA misincorporation can lead to a G to T transversion in subsequent rounds of replication. To counter this, cellular replisomes use specific TLS polymerases that preferentially incorporate dCTP opposite 8-oxo-dGMP.^56^ We show that apPol bypasses 8-oxo-dGMP with an almost equal propensity of incorporating a dCTP or a dATP opposite this lesion. This promiscuity, reflected in the low fidelity of 8-oxo-dG bypass (1.6; Table S2), is a distinguishing characteristic of apPol that is in contrast with other A-family polymerases studied to date. Although the accuracy of bypass varies between different A-family polymerases (Table S2), these polymerases preferentially incorporate dCTP opposite 8-oxo-dG.

Our results further show that the proofreading activity of apPol cannot discriminate between dCMP:8-oxo-dGMP and dAMP:8-oxo-dGMP base pairs, treating them instead as mispairs to be removed by the exonuclease site, suggesting that even the “correct” dCMP:8-oxo-dGMP pair does not adopt the canonical Watson–Crick geometry. Our observations are consistent with previous structural studies investigating the molecular mechanism of 8-oxo-dGMP bypass by the A-family *Bacillus stearothermophilus* DNA polymerase I,^57^ where the authors showed that a dCMP:8-oxo-dGMP base pair in a postinsertion binary complex resembles a DNA mismatch. Unlike the bypass of an abasic site, apPol bypasses 8-oxo-dGMP with relatively high efficiency (Table S2), making it possible that a significant fraction of 8-oxo-dGMP lesions is bypassed by apPol during apicoplast DNA replication. Given the low fidelity of apPol’s nucleotide incorporation opposite this lesion, such bypass might be a major source of mutations within the apicoplast genome. For viral and cellular replisomes from all domains of life, it has been reported that partner proteins such as the processivity factor can increase the fidelity and/or lesion bypass activity of a DNA polymerase,^58−63^ and it remains to be seen if this holds true for apPol-mediated TLS.

### Presteady-State Assays Recapitulate the Kinetic Mechanism of Processive Synthesis

While this work reports the first transient state kinetic analysis of nucleotide incorporation and TLS activities of apPol, there have been previous reports on the multiple turnover kinetics of apPol, including a recent study exploring the TLS capacity of this polymerase (albeit with a slightly different construct).^8,14,15^ Based on the steady-state analysis, the authors concluded that the rate of dNTP incorporation opposite an undamaged base by apPol is the same as the rate of adding a nucleotide opposite an oxidative lesion.^15^ This conclusion is inconsistent with our results and arises from the limitations of steady-state primer extension assays. We found that apPol copies an undamaged nucleotide over an order of magnitude faster compared to replicating 8-oxo-dGMP (Tables S1 and S2). In fact, the rates of nucleotide incorporation determined by us are over 1 to 3 orders of magnitude faster than the corresponding steady-state rates reported previously.^8,14,15^ Moreover, the efficiencies of incorporation of dCTP and dATP opposite 8-oxo-dGMP calculated in this work are an order of magnitude higher than the corresponding efficiencies reported based on steady-state experiments.^15^

Single nucleotide incorporation assays performed under multiple turnover conditions are dominated by the rate-limiting step, which, for most DNA polymerases including apPol, is the dissociation of the postchemistry binary complex.^64^ Consequently, steady-state assays primarily report on DNA dissociation and not on the bond formation and prechemistry ternary complex formation steps. However, these two steps are the main determinants of a polymerase’ efficiency and fidelity. Moreover, as described before, in vivo a polymerase performs processive synthesis by translocating along the DNA rather than dissociating and rebinding (Figure 1B), making steps 1 and 5 of binary complex formation and dissociation irrelevant during processive synthesis. Therefore, although steady-state experiments remain an excellent starting point for the preliminary characterization of a DNA polymerase, the kinetic information derived from these assays typically do not represent the kinetics followed by this enzyme in vivo and frequently underestimate the effects of altered substrates on polymerase kinetics.^18^

## Conclusions and Future Directions

Owing to apPol’s putative function as the only apicoplast polymerase, inhibiting polymerase activity has been proposed to be a viable approach to treat malaria,^12,65,66^ and the kinetic characterization presented here lays down the foundation for future structure–function correlation studies with this therapeutically relevant enzyme. Each reaction intermediate described in the catalytic cycle of apPol here represents a potential drug target. However, to effectively target the functional intermediates, further structural understanding of the substrate- and product-bound forms of apPol is imperative. Characterization of viral, bacterial, and eukaryotic replisomes has proven invaluable for understanding the similarities and diversity in how replication is coordinated within different systems.^67−69^ Future work focusing on the entire apicoplast replisome will be crucial for elucidating how this nanomachine copies the A/T-rich, repetitive apicoplast genome with high speed and accuracy.

## Supporting Information Available

Comparison of the kinetic parameters of apPol with other replicative TLS and repair DNA polymerases; comparison of the fidelity of 8-oxo-dGMP bypass by apPol with the corresponding fidelities of other A-family DNA polymerases; kinetics of correct dNTP incorporation by apPol using nonlinear regression; 1D and 2D FitSpace analysis of the global kinetic data fitting; and analysis of the lesion bypass kinetics of apPol using nonlinear regression (PDF)

## Notes

The mechanism files for the global fitting are available upon request.

## Notes

UniProt ID of the protein under study Q8ILY1 · Q8ILY1_PLAF7.
