# Supplementary material for: Transient State Kinetics of Plasmodium falciparum Apicoplast DNA Polymerase Suggests the Involvement of Accessory Factors for Efficient and Accurate DNA Synthesis
Source: Biochemistry. 2022 Oct 17;61(21):2319–33. doi: 10.1021/acs.biochem.2c00446 (PMC9631997; doi:10.1021/acs.biochem.2c00446)
Supplement: Supplementary file 1 — bi2c00446_si_001.pdf [file bi2c00446_si_001.pdf]

Transient state kinetics of *Plasmodium falciparum* apicoplast DNA polymerase suggests the involvement of accessory factors for efficient and accurate DNA synthesis

Anamika Kumari[1], Anjali Yadav[1], Indrajit Lahiri[1,2*]

[1] Department of Biological Sciences, Indian Institute of Science Education and Research Mohali, Punjab 140306, India
[2] Molecular Microbiology, School of Biosciences, University of Sheffield, Sheffield S10 2TN, UK

*** Contact information**

Indrajit Lahiri   I.Lahiri@sheffield.ac.uk

DNA Polymerase	K_D^{DNA} (nM)	K_D^{dNTP} (μM)	k_{pol} (s^{-1})	k_{off} (s^{-1})	Processivity (k_{pol}/k_{off})	Efficiency (k_{pol}/K_D^{dNTP}) ($\mu M^{-1}s^{-1}$)	Ref.
P. falciparum* apPol	78	131	30	1.9	16	0.23	This study
**T7 Pol- thioredoxin***	18	18	287	0.2	1435	16	1
**Human Pol gamma holoenzyme***	9.9	0.78	45	0.02	2250	58	2
**Mammalian Pol delta-PCNA***	64	0.93	21	0.006	3500	22.6	3
S. cerevisiae* Pol epsilon holoenzyme	22	11	411	0.0058	70862	37.4	4
***E. coli* Pol IV§**	50	441	12	0.18	67	0.03	5
**Rat Pol beta****	49	110	10	0.3	163	0.09	6

Table S1. Kinetic parameters for nucleotide incorporation opposite undamaged dNMP by different DNA polymerases. *: replicative polymerase, §: TLS polymerase: **: DNA repair polymerase.

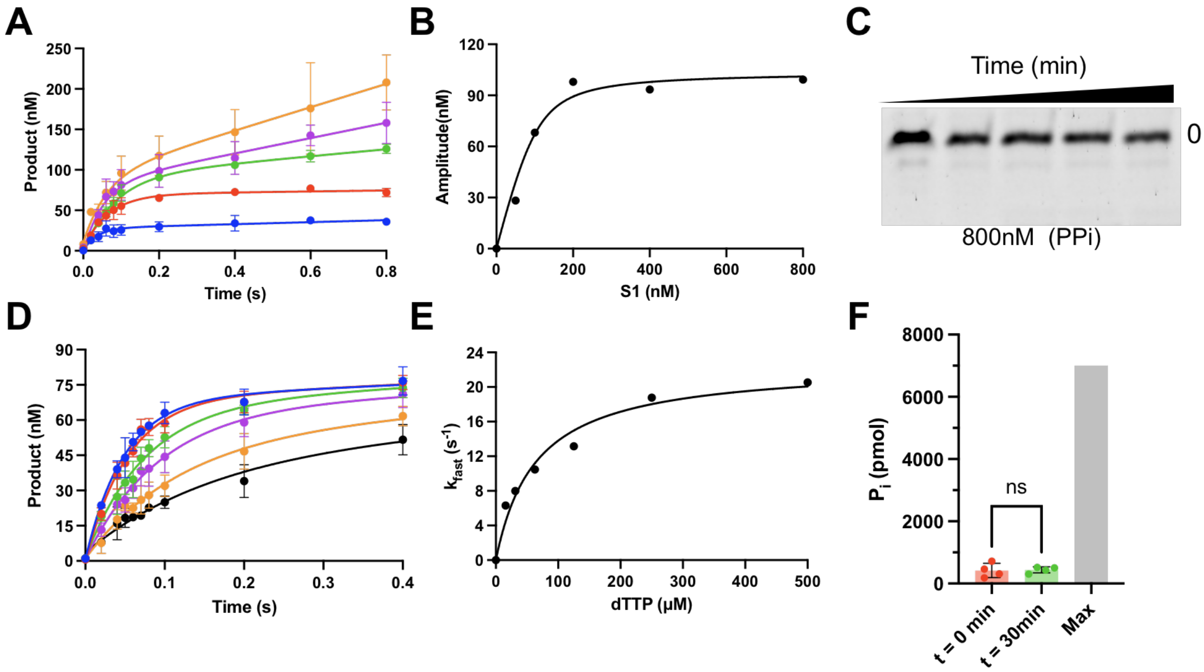

Figure S1. Kinetics of correct dNTP incorporation by apPol opposite an undamaged dNMP. (A and B) Active site titration of apPol analysed by non-linear regression. (**A)** Time courses shown in Fig.2C fit to the full burst equation. The assays were performed as described in the main text. **(B)** The amplitudes of the fast exponential phases of product formation plotted as a function of DNA substrate S1 concentration. The data was fit to a quadratic equation and from the fit $K_{D,app}^{DNA}$ was calculated to be 17.8 ± 9 nM and the active fraction of apPol was 104 ± 6.4 nM, which is ~ 22% of the total apPol (480 nM) added. **(C)** Acrylamide-urea gel depicting primer degradation assay in the presence of 800 mM PP_i. A final concentration of 104 nM active apPol was incubated with 100 nM of DNA substrate S1 and 800 nM PP_i in apPol reaction buffer. The reactions were incubated at 37˚C for various time intervals ranging from 0 to 2 min (0, 0.33, 0.5, 1 and 2 min) and then quenched with excess EDTA. The samples were then analysed on a 15% acrylamide-urea denaturing gel. 0: undegraded primer strand. **(D and E)** Single nucleotide primer extension assays for determining the affinity of dNTP for the pre-chemistry binary complex and the rate of bond formation. **(D)** Time courses shown in Fig.2E fit to the full burst equation. The assays were performed as described in the main text. **(E)** The rates of the fast phase of the the time courses from panel D (k_{fast}) plotted as a function of dTTP concentration. The data was fit to the hyperbolic equation and from the fit maximal rate of bond formation (k_{pol}) was calculated to be 22.6 ± 1.6 s^{-1} and the apparent dissociation constant for nucleotide binding ($K_{D,app}^{dNTP}$) was 64.2 ± 14.3 µM. **(F)** Detection of the amount of P_i released during replication of the M13mp18 single stranded (ss) DNA by apPol. The red bar (t = 0 min) shows the amount of phosphate present in the assay before the start of the polymerization reaction. The green bar (t = 30 min) shows the amount of phosphate generated after 30 minutes of the polymerization reaction. The grey bar (Max) shows the maximum amount of phosphate expected to be generated if P_i was the product released from the post-chemistry complex. The experiments were performed four times and the average of the four data sets are plotted as translucent bars while the error bars represent the SD of the data. ns: Not significant based on paired parametric t test.

A

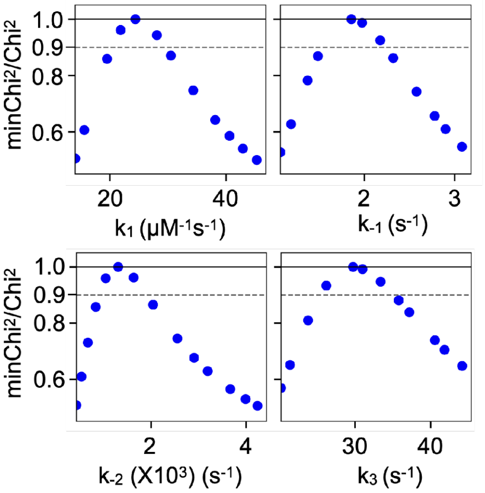

B

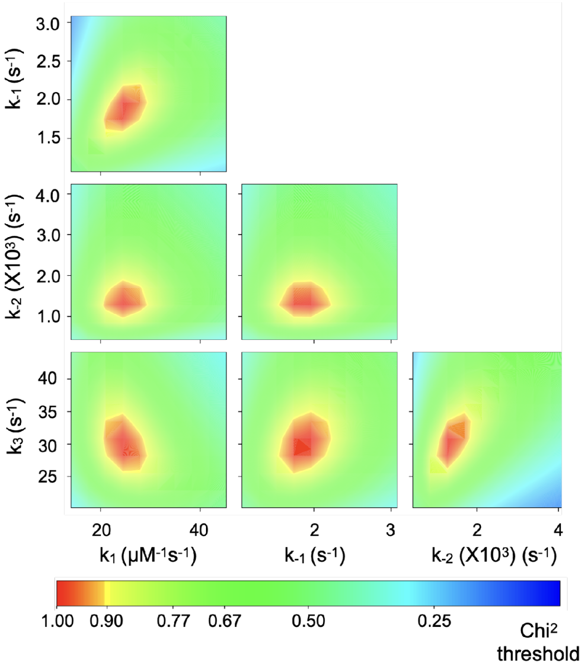

C

Rate constant	Best fit	Standard error	Lower bound	Upper bound
k_1 ($\mu M^{-1}s^{-1}$)	24.4	1.1	21	28.1
k_{-1} (s^{-1})	1.86	0.9	1.7	2.2
k_{-2} (s^{-1})	1307	114	1050	1700
k_3 (s^{-1})	30	1.2	25.5	33.5

Figure S2. Confidence contour analysis for the kinetics of correct nucleotide incorporation opposite undamaged template by apPol. (**A**) One dimensional confidence contour analysis of the rate constants governing the kinetic scheme shown in Figure 2B. The forward rate constant for nucleotide binding was fixed at a diffusion limited rate constant of 10 $\mu M^{-1}s^{-1}$. The dashed lines represent the minimum chi^2/chi^2 threshold value of 0.9 (recommended by the FitSpace routine in KinTek Explorer) based on which the lower and upper bounds of the rate constants were determined. A ratio of 1 represent the best-fit value. (**B**) Two dimensional confidence contour analysis of the rate constants governing the kinetic scheme shown in Figure 4A. The heat maps display the confidence intervals at the indicated chi^2 thresholds for each pairs of rate constants. (**C**) Table listing the best fit values, standard errors and the upper and lower bounds (determined from the 1D and 2D confidence contour analysis) of the rate constants governing the kinetic pathway shown in Fig.2B.

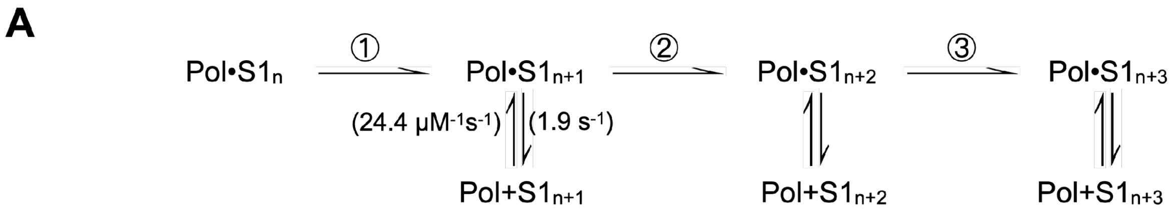

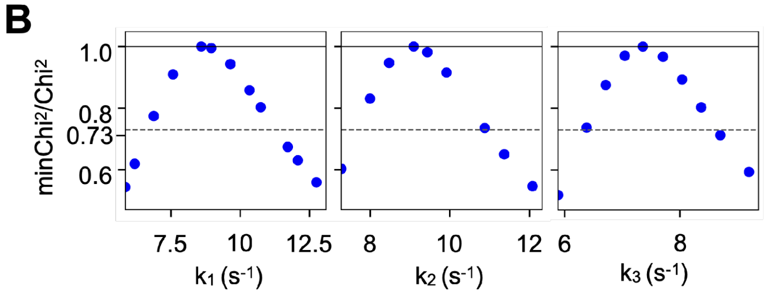

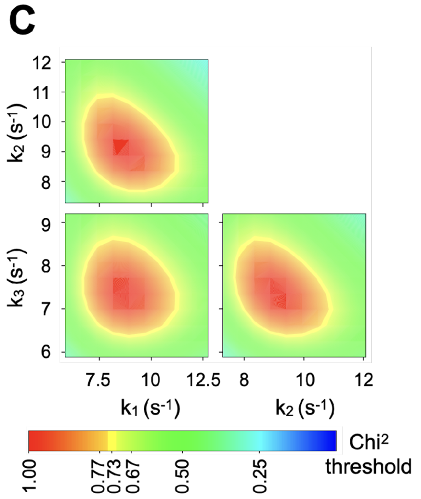

D

Rate constant	Best fit	Standard error	Lower bound	Upper bound
k_1 (s^{-1})	8.6	1.1	6.7	11.2
k_2 (s^{-1})	9.1	0.8	7.8	11
k_3 (s^{-1})	7.4	0.6	6.4	8.5

Figure S3. Confidence contour analysis for the kinetics of processive synthesis on undamaged template by apPol. (**A**) Kinetic scheme used for global fitting of the time courses shown in Fig.4B. The forward and reverse rate constants governing the formation of the binary complexes were fixed at 24.4 $\mu M^{-1}s^{-1}$ and 1.9 s^{-1} respectively, based on the corresponding rate constants determined from the single nucleotide incorporation kinetics of apPol (Figs.2 and S2). For clarity, the fixed rate constants are shown (in parenthesis) for only one binary complex (Pol•$S1_{n+1}$) formation step. $S1_n$: Substrate S1 with 23 nucleotide long unextended primer strand. $S1_{n+1}$, $S1_{n+2}$, $S1_{n+3}$: Substrate S1 with primer strand extended by one nucleotide, two and three nucleotides respectively. (**B**) One dimensional confidence contour analysis of the rate constants governing the kinetic scheme shown in panel A. The dashed lines represent the minimum chi^2/chi^2 threshold value of 0.73 (recommended by the FitSpace routine in KinTek Explorer) based on which the lower and upper bounds of the rate constants were determined. A ratio of 1 represent the best-fit value. (**C**) Two dimensional confidence contour analysis of the rate constants governing the kinetic scheme shown in panel A. The heat maps display the confidence intervals at the indicated chi^2 thresholds for each pairs of rate constants. (**D**) Table listing the best fit values, standard errors and the upper and lower bounds (determined from the 1D and 2D confidence contour analysis) of the rate constants governing the kinetic pathway shown in panel A.

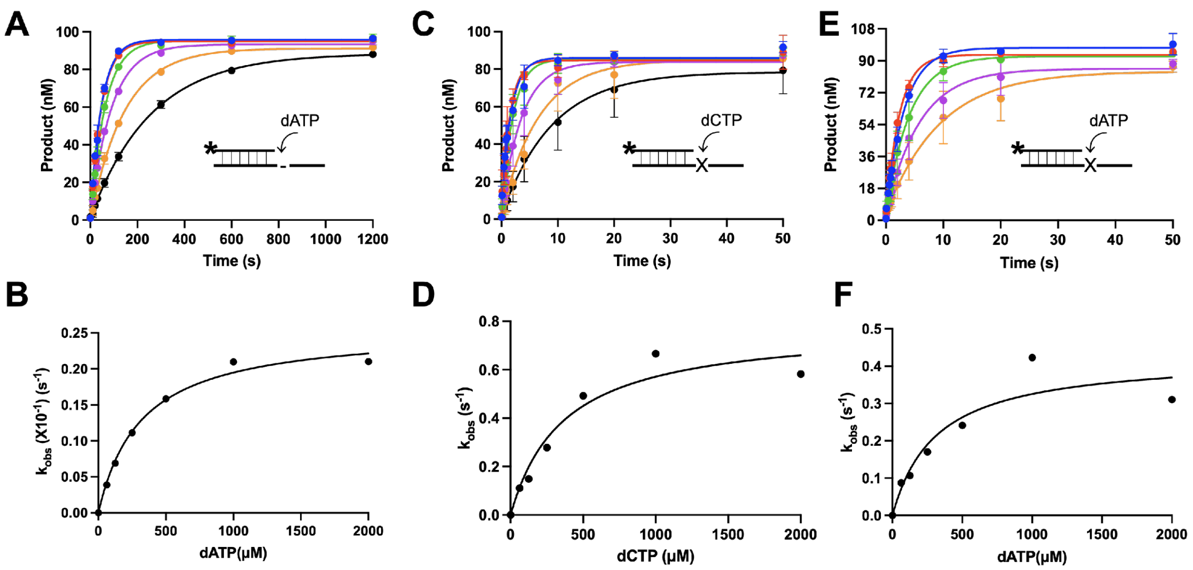

Figure S4. Analysis of TLS by apPol using traditional fit. (**A**) The time courses of dATP incorporation opposite an abasic site at varying dATP concentrations (shown in Figure 6B) fitted to the single exponential equation. The colour scheme is the same as that in Figure 6B. (**B**) Rates of product formation (k_{obs}) determined from the time courses of panel A plotted as a function of dATP concentration. The data was fit to the hyperbolic equation and from there the maximal rate of nucleotide incorporation (k_{pol}) was determined to be 0.026 ± 0.001 s^{-1} and the apparent K_D for dATP binding to the binary complex ($K_{D,app}^{dATP:abasic}$) was 316 ± 44 µM. (**C**) The time courses of dCTP incorporation opposite 8-oxo-dGMP at varying nucleotide concentrations (shown in Figure 7B) fitted to the single exponential equation. The colour scheme is the same as that in Figure 7B. (**D**) Rates of product formation (k_{obs}) determined from the time courses of panel C plotted as a function of dCTP concentration. The data was fit to the hyperbolic equation and from there the maximal rate of nucleotide incorporation (k_{pol}) was determined to be 0.78 ± 0.09 s^{-1} and the apparent K_D for dCTP binding to the binary complex ($K_{D,app}^{dCTP:8oxodG}$) was 367 ± 130 µM. (**E**) The time courses of dATP incorporation opposite 8-oxo-dGMP at varying nucleotide concentrations (shown in Figure 7C) fitted to the single exponential equation. The colour scheme is the same as that in Figure 7C. (**F**) Rates of product formation (k_{obs}) determined from the time courses of panel D plotted as a function of dATP concentration. The data was fit to the hyperbolic equation and from there the maximal rate of nucleotide incorporation (k_{pol}) was determined to be 0.43 ± 0.07 s^{-1} and the apparent K_D for dATP binding to the binary complex ($K_{D,app}^{dATP:8oxodG}$) was 312 ± 160 µM. **Panels A,C and E Insets**: Schematics of the reactants used for the respective primer extension assays. All the experiments were performed in triplicate and the average of the three independent data sets are plotted in the graphs shown in panels A,C and E while the error bars represent the standard deviation (SD) of data sets. ± : standard error (SE).

A

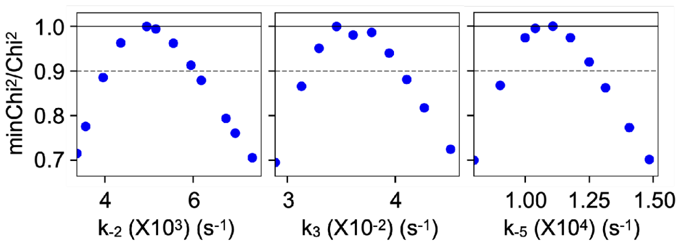

B

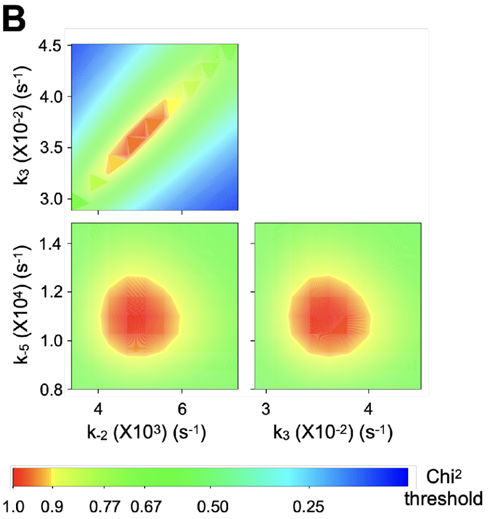

C

Rate constant	Best fit	Standard error	Lower bound	Upper bound
k_{-2} (s^{-1})	4946	177	4170	5760
k_3 (s^{-1})	0.036	0.002	0.03	0.04
k_{-5} (s^{-1})	-	-	9520	12600
k_6 (s^{-1})	0.001	5.2X10^{-5}	-	-

Figure S5. Confidence contour analysis for the kinetics of abasic site bypass by apPol. (**A**) One dimensional confidence contour analysis of the rate constants governing the kinetic scheme shown in Fig.6C. The forward rate constants for nucleotide binding were fixed at a diffusion limited rate constant of 10 $\mu M^{-1}s^{-1}$ and the forward and reverse rate constants governing the binding of apPol to the DNA substrates were fixed at 24.4 $\mu M^{-1}s^{-1}$ and 1.9 s^{-1} respectively (best-fit values derived from the kinetics of single nucleotide incorporation opposite an undamaged template (Fig.2)). The dashed lines represent the minimum chi^2/chi^2 threshold value of 0.9 (recommended by the FitSpace routine in KinTek Explorer) based on which the lower and upper bounds of the rate constants were determined. A ratio of 1 represent the best-fit value. (**B**) Two dimensional confidence contour analysis of the rate constants governing the kinetic scheme shown in Figure 6C. The heat maps display the confidence intervals at the indicated chi^2 thresholds for each pairs of rate constants. (**C**) Table listing the best fit values, standard errors and the upper and lower bounds (determined from the 1D and 2D confidence contour analysis) of the rate constants governing the kinetic pathway shown in Fig.6C. The primer extension data shown in Figs.6 B,D and E could not constrain both k_6 and k_{-5} independently. However, the ratio of the two rate constants were well-constrained. Hence, during global fitting k_6 and k_{-5} were linked at their individual best-fit values and then the two rate constants were allowed to float as a pair. Thus, the best-fit value and SE of k_6 is shown in panel C and only k_{-5} was included in the confidence contour analysis shown in panels A and B.

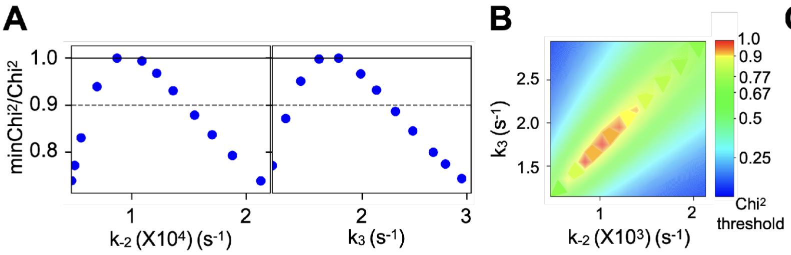

C

Rate constant	Best fit	Standard error	Lower bound	Upper bound
k_{-2} (s^{-1})	8717	1831	6580	13900
k_3 (s^{-1})	1.6	0.2	1.35	2.15

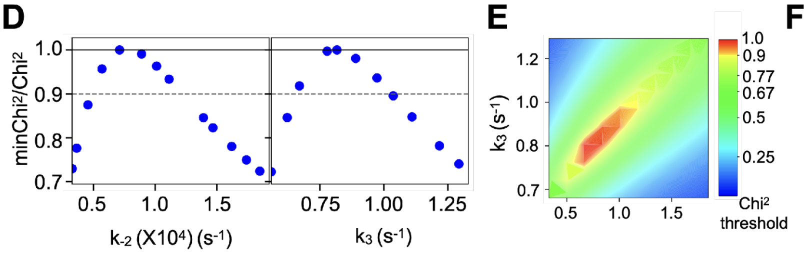

F

Rate constant	Best fit	Standard error	Lower bound	Upper bound
k_{-2} (s^{-1})	7127	1251	5340	11700
k_3 (s^{-1})	0.78	0.06	0.67	0.97

Figure S6. Confidence contour analysis for the kinetics of 8-oxo-dGMP bypass by apPol. (**A**) One dimensional confidence contour analysis of the rate constants governing the kinetics of dCTP incorporation opposite 8-oxo-dGMP (Figure 7B). The kinetic scheme is shown in Figure 7D. The forward rate constants for nucleotide binding were fixed at a diffusion limited rate constant of 10 $\mu M^{-1}s^{-1}$ and the forward and reverse rate constants governing the binding of apPol to the DNA substrates were fixed at 24.4 $\mu M^{-1}s^{-1}$ and 1.9 s^{-1} respectively (best-fit values derived from the kinetics of single nucleotide incorporation opposite an undamaged template (Fig.2)). The dashed lines represent the minimum chi^2/chi^2 threshold value of 0.9 (recommended by the FitSpace routine in KinTek Explorer) based on which the lower and upper bounds of the rate constants were determined. A ratio of 1 represent the best-fit value. (**B**) Two dimensional confidence contour analysis of the rate constants governing dCTP incorporation opposite 8-oxo-dGMP. The heat maps display the confidence intervals at the indicated chi^2 thresholds for the pair of rate constants. (**C**) Table listing the best fit values, standard errors and the upper and lower bounds (determined from the 1D and 2D confidence contour analysis) of the rate constants governing the kinetic pathway of dCTP incorporation opposite 8-oxo-dGMP. (**D**) One dimensional confidence contour analysis of the rate constants governing the kinetics of dATP incorporation opposite 8-oxo-dGMP (Figure 7C). The kinetic scheme is shown in Fig.7D. The forward rate constants for nucleotide binding were fixed at a diffusion limited rate constant of 10 $\mu M^{-1}s^{-1}$ and the forward and reverse rate constants governing the binding of apPol to the DNA substrates were fixed at 24.4 $\mu M^{-1}s^{-1}$ and 1.9 s^{-1} respectively (best-fit values derived from the kinetics of single nucleotide incorporation opposite an undamaged template (Fig.2)). The dashed lines represent the minimum chi^2/chi^2 threshold value of 0.9 (recommended by the FitSpace routine in KinTek Explorer) based on which the lower and upper bounds of the rate constants were determined. A ratio of 1 represent the best-fit value. (**E**) Two dimensional confidence contour analysis of the rate constants governing dATP incorporation opposite 8-oxo-dGMP. The heat maps display the confidence intervals at the indicated chi^2 thresholds for the pair of rate constants. (**F**) Table listing the best fit values, standard errors and the upper and lower bounds (determined from the 1D and 2D confidence contour analysis) of the rate constants governing the kinetic pathway of dATP incorporation opposite 8-oxo-dGMP.

DNA Polymerase	$K_D^{dCTP:8oxodG}$ (μM)	$k_{pol}^{dCTP:8oxodG}$ (s^{-1})	$Efficiency^{dCTP:8oxodG}$ ($\mu M^{-1}s^{-1}$)	$K_D^{dATP:8oxodG}$ (μM)	$k_{pol}^{dATP:8oxodG}$ (s^{-1})	$Efficiency^{dATP:8oxodG}$ ($\mu M^{-1}s^{-1}$)	Fidelity	$Efficiency^{Ud}$ ($\mu M^{-1}s^{-1}$)	Ref.
***P. falciparum* apPol**	872	1.6	0.0018	713	0.78	0.0011	1.6	0.23	This study
**T7 Pol-thioredoxin**	11	1.6	0.14	110	0.2	0.0018	78	27.6	7
**Human Pol gamma holoenzyme**	100	10	0.1	300	3.4	0.011	9	40.8	8
***E. coli* Pol I**	19	5.5	0.3	190	0.3	0.0016	187.5	10.3	9

Table S2. Kinetic parameters for 8-oxo-dGMP bypass by different A-family DNA polymerases. $Efficiency^{dCTP:8oxodG} = (k_{pol}^{dCTP:8oxodG}/K_D^{dCTP:8oxodG})$, $Efficiency^{dATP:8oxodG} = (k_{pol}^{dATP:8oxodG}/K_D^{dATP:8oxodG})$, Fidelity = $(Efficiency^{dCTP:8oxodG}/ Efficiency^{dATP:8oxodG})$, $Efficiency^{Ud}$: Efficiency of nucleotide incorporation opposite undamaged dNMP.

References

(1) Patel, S. S., Wong, I., and Johnson, K. A. (1991) Pre-steady-state kinetic analysis of processive DNA replication including complete characterization of an exonuclease-deficient mutant. *Biochemistry 30*, 511–525.

(2) Johnson, A. A., Tsai, Y. c, Graves, S. W., and Johnson, K. A. (2000) Human mitochondrial DNA polymerase holoenzyme: reconstitution and characterization. *Biochemistry 39*, 1702–1708.

(3) Einolf, H. J., and Guengerich, F. P. (2000) Kinetic analysis of nucleotide incorporation by mammalian DNA polymerase delta. *J. Biol. Chem. 275*, 16316–16322.

(4) Zahurancik, W. J., and Suo, Z. (2020) Kinetic investigation of the polymerase and exonuclease activities of human DNA polymerase ε holoenzyme. *J. Biol. Chem. 295*, 17251–17264.

(5) Bertram, J. G., Bloom, L. B., O'Donnell, M., and Goodman, M. F. (2004) Increased dNTP binding affinity reveals a nonprocessive role for Escherichia coli beta clamp with DNA polymerase IV. *J. Biol. Chem. 279*, 33047–33050.

(6) Werneburg, B. G., Ahn, J., Zhong, X., Hondal, R. J., Kraynov, V. S., and Tsai, M. D. (1996) DNA polymerase beta: pre-steady-state kinetic analysis and roles of arginine-283 in catalysis and fidelity. *Biochemistry 35*, 7041–7050.

(7) Furge, L. L., and Guengerich, F. P. (1997) Analysis of nucleotide insertion and extension at 8-oxo-7,8-dihydroguanine by replicative T7 polymerase exo- and human immunodeficiency virus-1 reverse transcriptase using steady-state and pre-steady-state kinetics. *Biochemistry 36*, 6475–6487.

(8) Hanes, J. W., Thal, D. M., and Johnson, K. A. (2006) Incorporation and replication of 8-oxo-deoxyguanosine by the human mitochondrial DNA polymerase. *J. Biol. Chem. 281*, 36241–36248.

(9) Lowe, L. G., and Guengerich, F. P. (1996) Steady-state and pre-steady-state kinetic analysis of dNTP insertion opposite 8-oxo-7,8-dihydroguanine by Escherichia coli polymerases I exo- and II exo-. *Biochemistry 35*, 9840–9849.
